# Amplification of oxidative stress with lycorine and gold-based nanocomposites for synergistic cascade cancer therapy

**DOI:** 10.1186/s12951-021-00933-1

**Published:** 2021-07-27

**Authors:** Hongzhi Hu, Wenbo Yang, Zihui Liang, Zezhu Zhou, Qingcheng Song, Weijian Liu, Xiangtian Deng, Jian Zhu, Xin Xing, Binglong Zhong, Baichuan Wang, Shangyu Wang, Zengwu Shao, Yingze Zhang

**Affiliations:** 1 Department of Orthopaedics, Union Hospital, Tongji Medical College, Huazhong University of Science and Technology, Wuhan, 430022 China; 2 Collaborative Innovation Center for Advanced Organic Chemical Materials Co-Constructed By the Province and Ministry, Hubei University, Wuhan, 430062 China; 3 Department of Orthopaedic Surgery, The Third Hospital of Hebei Medical University, Shijazhuang, 050051 China; 4 School of Medicine, Nankai University, Tianjin, 300071 China

**Keywords:** Oxidative stress, Mitochondrial dysfunction, Endoplasmic reticulum stress, Osteosarcoma, Lycorine, Gold nanostars, Mesoporous silica

## Abstract

**Background:**

Despite advances of surgery and neoadjuvant chemotherapy during the past few decades, the therapeutic efficacy of current therapeutic protocol for osteosarcoma (OS) is still seriously compromised by multi-drug resistance and severe side effects. Amplification of intracellular oxidative stress is considered as an effective strategy to induce cancer cell death. The purpose of this study was to develop a novel strategy that can amplify the intracellular oxidative stress for synergistic cascade cancer therapy.

**Methods and results:**

A novel nanocomposite, composed of folic acid (FA) modified mesoporous silica–coated gold nanostar (GNS@MSNs-FA) and traditional Chinese medicine lycorine (Ly), was rationally designed and developed. Under near-infrared (NIR) irradiation, the obtained GNS@MSNs-FA/Ly could promote a high level of ROS production via inducing mitochondrial dysfunction and potent endoplasmic reticulum (ER) stress. Moreover, glutathione (GSH) depletion during ER stress could reduce ROS scavenging and further enable efficient amplification of intracellular oxidative stress. Both in vitro and in vivo studies demonstrated that GNS@MSNs-FA/Ly coupled with NIR irradiation exhibited excellent antitumor efficacy without noticeable toxicity in MNNG/HOS tumor-bearing mice.

**Conclusion:**

All these results demonstrated that GNS@MSNs-FA/Ly coupled with NIR irradiation could dramatically amplify the intra-tumoral oxidative stress, exhibiting excellent antitumor ability without obvious systemic toxicity. Taken together, this promising strategy provides a new avenue for the effective cancer synergetic therapy and future clinical translation.

**Supplementary Information:**

The online version contains supplementary material available at 10.1186/s12951-021-00933-1.

## Introduction

Osteosarcoma (OS) is the most aggressive bone malignancy in children and adolescents, resulting in significant morbidity and mortality [1]. Despite advances of surgery and neoadjuvant chemotherapy during the past few decades, the overall survival rates of OS have reached a plateau [2]. However, these treatments still leave much to be improved, such as multi-drug resistance and severe side effects [3]. Therefore, there is an urgent need for the development of novel therapeutic strategies with high specificity and low toxicities to improve survival in patients with OS.

Emerging evidence implicates that traditional Chinese medicine (TCM) may be a valuable resource for the treatment of various cancers [4–6]. TCM have been shown to possess distinct advantages over commonly used chemotherapy medicine, such as low cost, high stability and minimal side effects [7, 8]. Lycroine (Ly), a natural active alkaloid, exhibits a wide range of pharmacological effects [9–11], including an excellent antitumor effect on various cancers [4, 12–14]. Our previous work [14] and other two studies [4, 15] have already confirmed that Ly possess remarkable therapeutic potential for OS. Studies suggested that antitumor effects of Ly might be associated with mitochondrial dysfunction in various types of cancer [12, 15, 16], including OS [15].

Mitochondria, as so-called cell powerhouses, are the main intracellular sources of reactive oxygen species (ROS) [17]. Excessive ROS production, known as oxidative stress, can lead to oxidative damage and reduced mitochondrial energy production efficiency, which further increases ROS generation and causes mitochondrial damage to form a “vicious circle” [12, 18, 19]. However, cancer cells normally possess a powerful antioxidant system to balance the increased level of intracellular ROS [20]. Therefore, disruption of intracellular redox homeostasis by increasing the ROS burden but also inhibiting the ROS scavenging could effectively amplify intracellular oxidative stress, so as to enable more effective cancer therapy [21].

As a player in the redox balance, endoplasmic reticulum (ER), exerts an essential role in protein synthesis/folding [22], which is prone to be influenced by extracellular stimuli and changes in intracellular homeostasis [23]. Studies indicated that any alteration of redox homeostasis in the ER would induce ER stress, which could, in turn, promote the generation of ROS [17, 22]. In addition, glutathione (GSH), the main ROS scavenger in cells, might be depleted during ER stress [24]. Therefore, promoting ER stress may lead to increased ROS production and GSH depletion. Recent findings showed that metal-based nanoparticles (NPs), such as silver NPs [25], gold NPs [26], iron oxide NPs [27], et al., could cause cell death through the induction of ER stress [23]. Gold NPs (GNPs) are emerging as novel agents for cancer treatment due to their excellent biocompatibility and molecular-recognition properties [26, 28]. In addition, GNPs have great potential for photothermal therapy (PTT) due to their high photothermal conversion efficiency [29]. PTT not only can cause irreversible damage to tumors through thermal ablation but also can aggrandize the permeability of the cell membrane, facilitating NPs intake of cancer cells, so as to enhance treatment effects [30]. Based on the above, we hypothesized that the integration of Ly and GNPs into one nanoplatform could boost the generation of ROS so as to achieve synergistic therapeutic effect for OS. However, the bare GNPs are prone to clustering and aggregation under near-infrared (NIR) laser irradiation, thus compromising the phototherapy efficiency in the NIR window [31–33]. To overcome this weakness, one promising strategy is the encapsulation of gold NPs into some hard substances like mesoporous silica NPs (MSNs) to construct core–shell structural nanocomposites [34, 35]. Of note, due to the high loading capacity [36] and easily functionalized surface [37], MSN as a promising drug delivery system is expected to circumvent limitations of Ly’s poor aqueous solubility and insufficient target specificity [38].

**Scheme 1:**
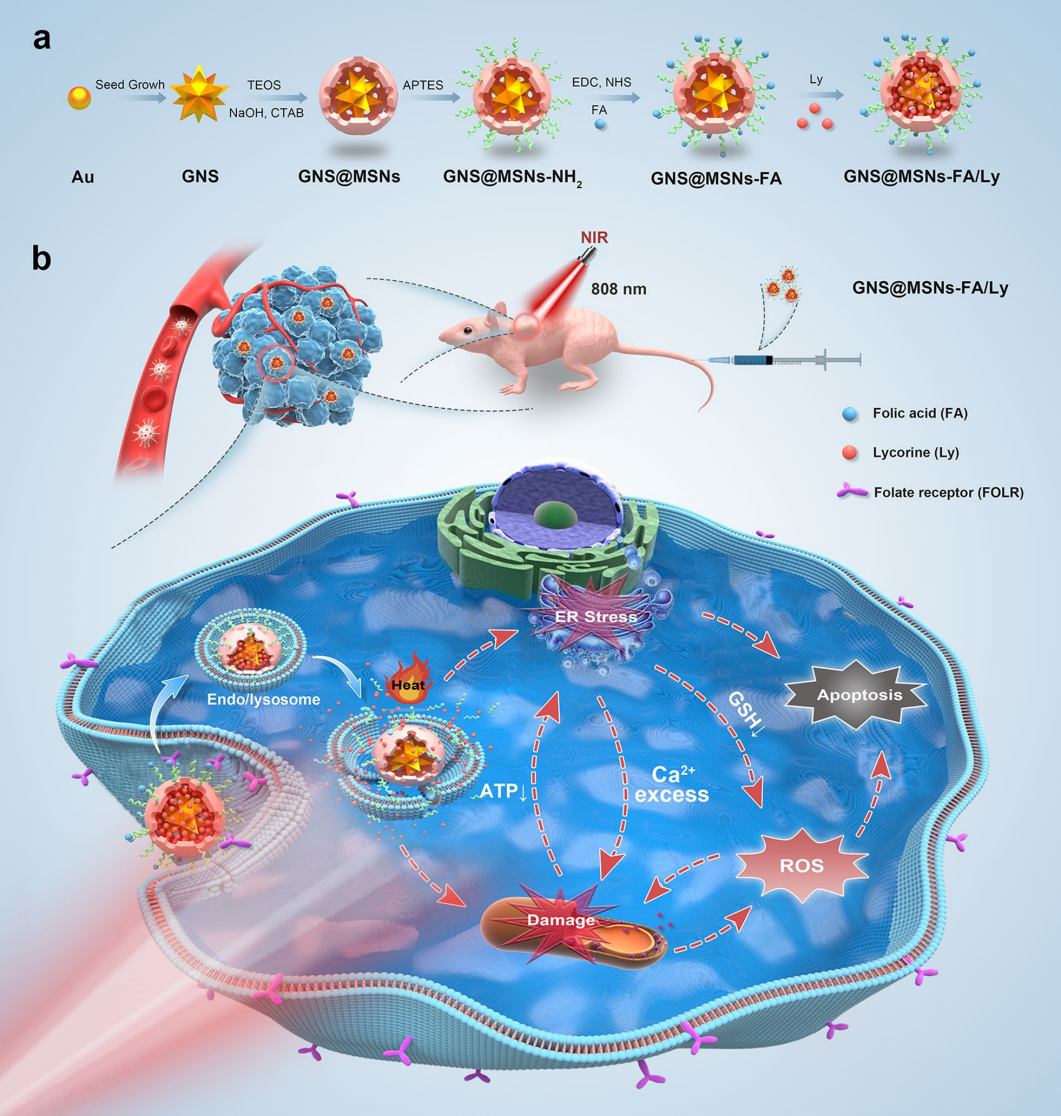
**a** Schematic illustration of the preparation process of GNS@MSNs-FA/Ly. **b** The therapy mechanism of GNS@MSNs-FA/Ly for synergistic cascade cancer therapy

Hereby, we rationally designed and successfully fabricated a gold-based nanocomposite (GNS@MSNs-FA/Ly) for synergistic cascade cancer therapy (Scheme 1a). Briefly, Gold nanostars (GNSs), with multiple sharp branches, were chosen to be the core of nanocomplex. Furthermore, the GNS core was coated with MSNs to form core–shell GNS@MSNs and then the surface of GNS@MSNs was modified with amino group. The folate receptor (FOLR) is a tumor-associated protein overexpressed on the surface of various cancer cells, so the modification of folic acid (FA) molecules can actively target them to tumor cells [39–41]. Thus, FA molecules with specific tumor-targeting properties were conjugated onto amine-functionalized GNS@MSNs (GNS@MSNs-NH_2_) via a dehydrative condensation reaction to form GNS@MSNs-FA. Finally, Ly was loaded into the porous MSNs through the physical adsorption. The synergistic cascade therapeutic properties of these multifunctional nanocomposites were further evaluated in vitro and validated in MNNG/HOS tumor-bearing mice model in vivo. After the nanocomposite was internalized into cancer cells through receptor-mediated endocytosis, the loaded Ly released explosively owing to the acidic tumor microenvironment and hyperthermia generated from NIR irradiation. Ly released in a burst caused mitochondrial dysfunction and concomitant mitochondrial ROS overproduction. Excess ROS could cause mitochondrial dysfunction, which in turn further promoted the ROS generation. The ATP shortage resulting from mitochondrial dysfunction and heat stress generated by PTT enhanced the ER stress caused by GNS. Moreover, the enhanced ER stress could lead to Ca^2+^ release and GSH depletion. All of these mechanisms ultimately impaired cellular homeostasis and induces cell apoptosis (Scheme 1b). Overall, this work provides a tempting strategy for future applications in synergistic treatment of malignant tumors.

## Material and methods

### Materials

Hydrogen tetrachloroaurate (HAuCl_4_), trisodium citrate, sodium borohydride (NaBH_4_), silver nitrate (AgNO_3_), ascorbic acid, cetyltrimethylammonium bromide (CTAB), (3-Aminopropyl) triethoxy silane (APTES) and ammonium nitrate (NH_4_NO_3_) were purchased from Aladdin-reagent. Sodium hydroxide (NaOH) and tetraethylorthosilicate (TEOS) were purchased from Sinopharm Chemical Reagent Co., Ltd, (Shanghai, China). N-(3-dimethylaminopropyl)-N-ethylcarbodiimide hydrochloride (EDC), N-hydroxysuccinimide (NHS), and folic acid (FA) were purchased from Sigma-Aldrich (St. Louis, MO, USA). Lycorine (Ly, purity > 98%), obtained from Solarbio (Beijing, China), was dissolved in dimethylsulfoxide (DMSO) (Sigma) and stored at − 20 °C. Primary antibodies against Bcl-2, Bax, Cytochrome c (Cyt-c), p-eIF2α, ATF4, CHOP, and GAPDH were purchased from GeneTex (Irvine, CA, USA). Other chemicals were commercially available and used without further purification.

### Synthesis of GNS

The GNS was prepared using a seed-mediated growth progress with slight modifications [42, 43]. Briefly, to prepare the seed solution, 1 mL trisodium citrate solution (38 mM), 0.13 mL HAuCl_4_ (0.1 M) and 44 mL deionized (DI) water were mixed at room temperature. After that, 50 μL fresh ice-cold NaBH_4_ (0.2 M) was injected into the mixture quickly under vigorous stirring to form gold seeds. For GNS synthesis, 2 mL of the gold seed solution was added under gentle stirring to 0.5 mL of HAuCl_4_ (0.1 M) solution, dissolving to 200 mL DI water. Following, 200 μL of 2.5 mM AgNO_3_ and 1 mL of 100 mM ascorbic acid solutions were added simultaneously. After 30 min of stirring, the color of the solution would turn from bright red to blue-black indicating the success of GNS synthesis, which was stored at 4 ºC for further use.

### Preparation of GNS@MSNs and GNS@MSNs-NH_2_

Modified Stöber method was applied to prepare mesoporous silica-coated GNS (GNS@MSNs) according to the protocol described elsewhere [44]. In brief, 100 mL of CTAB (2 mg mL^−1^) was added into the above GNS solution and stirred for 30 min at 28 °C. Next, a moderate amount of NaOH was added to adjust the pH value to about 11. Subsequently, 750 µL of TEOS was added into the solution dropwise under vigorous stirring, and the reaction was carried on at 70 °C for 3 h, resulting in the formation of GNS@MSNs solution. Then, 0.5 mL APTES was added to the solution and gently stirred for 5 h at room temperature. The mixture was centrifuged and washed with ethanol for 3 times. Finally, the CTAB template was completely removed by dispersing the precipitate in 50 mL of 10 mg mL^−1^ NH_4_NO_3_ ethanol solution and washed in ethanol under reflux for 4 h, which was repeated for 3 times. The final products were denoted as amine-functionalized GNS@MSNs (GNS@MSNs-NH_2_) and dried with lyophilization.

### Synthesis of GNS@MSNs-FA

FA molecules were conjugated to the GNS@MSNs-NH_2_ according to the literature with slight modifications [45]. Briefly, 2 mg FA, 100 mg EDC and 50 mg NHS were dissolved in 2 mL DMSO, respectively. The three solutions were then mixed and stirred for 3 h to activate the carboxyl groups of FA. The activated FA was dropwise added into the above DMSO solution of GNS@MSNs-NH_2_ (2 mg mL^−1^, 4 mL) under gentle stirring in the dark for 24 h at room temperature. After that, the solution was dialyzed against DI-water to remove the excess reactants (EDC, NHS and FA). Finally, the solution was dried with lyophilization (denoted as GNS@MSNs-FA).

### Loading Ly on GNS@MSNs-FA

The GNS@MSNs-FA (5 mg) were first dispersed in Ly (2 mg/mL in DMSO, 5 mL) by ultrasonication. Next, the mixtures were gently stirred at room temperature for 24 h. Subsequently, the Ly-loaded GNS@MSNs-FA (GNS@MSNs-FA/Ly) was collected by centrifugation at 15,000 rpm for 5 min and washed 3 times with DMSO to remove the unbound drug. The yielded NPs were then washed with PBS for 3 times and stored at 4 °C before further use.

To evaluate the Ly loading content and entrapment efficiency, the standard curve of Ly was prepared with a UV–VIS spectrophotometer (Shimadzu UV3600). The supernatant and washed solutions were collected and the residual Ly content was determined according to the standard curve. The drug loading content and entrapment efficiency were calculated using the following equations [46]: Loading content % = (Weight of drug loaded into the NPs) /Weight of NPs + Weight of drug loaded into the NPs); Encapsulation efficiency % = Weight of drug loaded into the NPs / Initial weight of drug.

### Characterization

The size and shape of the NPs were observed by high-resolution transmission electron microscopy (HRTEM, FEI Talos F200X), operating at an accelerating voltage of 200 kV. The elemental components were analyzed by using an Energy-dispersive X-ray (EDX) analyzer as the TEM accessory. The samples were dispersed in DI water and then dropped on a carbon film supported on a copper grid. The zeta potential and size of samples were measured by using a Zetasizer Nano ZS90 (Malvern, UK). Fourier transform infrared spectra (FTIR) were scanned on a Thermo Nicolet iS50 FTIR spectrometer in the range of 400–4000 cm^−1^. The UV–Vis adsorption spectra was recorded using a spectrometer (UV-3600, Shimadzu, Japan).

### Photothermal performance of the materials

To measure the photothermal performance of GNS@MSNs-FA in vitro, the NPs were irradiated with the NIR laser equipment (Changchun Laser Optoelectronics Technology Co., Ltd.) with 808 nm wavelength and the temperature changes were monitored by using an infrared thermal imaging camera (Testo 865, Testo, Schwarzwald, Germany) in real-time. In brief, 200 μL GNS@MSNs-FA with various concentrations (25, 50, 100, and 200 μg mL^−1^, respectively) were placed on 96-well plates and irradiated with an 808 nm NIR laser at a power density of 1.0 W cm^−2^ for 5 min. Meanwhile, the PBS irradiated under the same conditions was used as a control. The temperature change was carefully measured and recorded every 30 s.

To further investigate the influence of the power density on photothermal heating, 100 μg mL^−1^ samples were illuminated at different power densities (0.5–2.0 W cm^−2^) and the change in temperature was recorded. In order to verify the thermal stability of GNS@MSNs-FA, the samples were irradiated with laser (1.0 W cm^−2^) for 5 min every time and then cooled to room temperature 3 times, and the temperature was carefully recorded every 30 s. Furthermore, the UV–vis absorbance values of GNS@MSNs-FA (100 μg mL^−1^) dispersed in PBS before and after 1 h of NIR laser irradiation (1.0 W cm^−2^) were measured to investigate the photothermal stability of GNS@MSNs-FA.

### pH- and photothermal-sensitive drug release profiles

The triggered release behavior of Ly from GNS@MSNs-FA/Ly NPs in different environments was investigated using the dialysis method. Briefly, 2 mg Ly-loaded NP suspension in 2 mL of PBS (pH 7.4) were first placed into a dialysis bag (MWCO = 3500 Da), and dialyzed against 50 mL of different buffer medium (pH 7.4 and pH 5.0) with or without exposure to 808 nm laser irradiation (1.0 W cm^−2^) for 5 min with gentle shaking at 37 °C. At specific intervals, 1.0 mL of release solution was taken out to quantify the amount of released drug using a UV–VIS spectrophotometer and flowed by compensation with an equal volume of fresh PBS to the sample solution.

### Hemolysis assay

Hemolytic activities of GNS@MSNs and GNS@MSNs-FA were evaluated by detecting the hemoglobin release from mice blood cells. Briefly, 2.0 mL anticoagulated whole blood samples obtained from healthy mice were centrifuged at 8000 rpm at 4 °C for 5 min and washed three times with PBS so as to obtain red blood cells (RBCs). The RBCs were diluted with 4.0 mL PBS. Subsequently, 0.8 mL of the PBS solutions of GNS@MSNs and GNS@MSNs-FA were added into 0.2 mL of diluted RBCs suspension, respectively, and the final concentrations of the NPs were 50, 100, 200, 400, 800, and 1600 µg mL^−1^. The negative and positive control groups were 0.2 mL of diluted RBC suspension plus 0.8 mL of PBS and deionized water, respectively. The mixed solutions were then incubated at 37 °C for 2 h in a shaker table. Subsequently, the mixtures were centrifuged at 8000 rpm at 4 °C for 5 min and the absorbance of the supernatant was determined with a UV–vis spectrophotometer at 541 nm. The following equation was used to calculate the hemolysis percentage of RBCs: Hemolysis percentages (%) = (OD_sample_ – OD_negative control_)/(OD_positive control_ – OD_negative control_) × 100%, where OD_sample_, OD_negative control_, OD_positive control_, and OD_negative control_ represent the absorbance of the sample, negative control, and positive control, respectively.

### Cell culture

Human OS cells (MNNG/HOS) were obtained from Cell Bank of Shanghai Institute of Biochemistry and Cell Biology, Chinese Academy of Sciences. The normal human cells, bone marrow stromal cells (BMSCs), were kindly provided by Dr. Song Gong (Tongji Medical College, Huazhong University of Science and Technology). The OS cells were routinely cultured and maintained in α‐modified essential medium (MEM) (Hyclone) containing 10% fetal bovine serum (FBS) (Gibco; Thermo Fisher Scientific), 1% antibiotics (penicillin and streptomycin). BMSCs were cultured with Dulbecco’s MEM (DMEM)/F12 containing 15% FBS and 1% penicillin–streptomycin. All cells were incubated at 37 °C in a humidified incubator with 5% CO_2_.

### Loading of indocyanine green (ICG)

10 mg NPs (GNS@MSNs or GNS@MSNs-FA) were dispersed in 5 mL deionized water and then 10 mg fluorescent dye ICG was added. The mixture was gently stirred for 24 h at room temperature. Subsequently, the product was washed three times with water and centrifuged at 8000 rpm for 10 min. After drying under vacuum, the fluorescence labeled materials (GNS@MSNs /ICG or GNS@MSNs-FA/ICG) were dispersed in PBS for further use.

### Cellular uptake

MNNG/HOS cells were seeded in six-well plates at a density of 1 × 10^5^ cells per well and grown overnight at 37 °C. Afterward, the cells were incubated with ICG-labeled materials (GNS@MSNs /ICG or GNS@MSNs-FA/ICG) at 37 °C in a humidified atmosphere of 5% CO_2_ for 4 h. Meanwhile, for competitive inhibition experiments, other two groups of cells were pre-incubated with 2 mM of FA at 37 °C for 2 h before the GNS@MSNs-FA/ICG was added. After that, the cells were washed carefully with PBS three times and the nucleus were stained with Hoechst 33,342 (10 μg mL^−1^) for 10 min. Finally, the cells were carefully washed and observed by using fluorescence microscopy (Olympus Corporation, Tokyo, Japan).

### Cell viability assay

Cell counting kit-8 (CCK-8, Dojindo, Kyushu Island, Japan) assay was performed to assess cell viability according to the manufacturer’s protocol. Briefly, cells were seeded in a 96-well plate at a density of 5000 cells per well and allowed to grow overnight. After the corresponding treatment, the cells were cultured at 37 °C in a humidified atmosphere of 5% CO_2_ for 24 h. Afterwards, the culture medium was replaced with 100 μL medium containing 10% CCK-8 solution and incubated in dark at 37 °C for 2 h. The absorbance of individual wells at 450 nm was measured by a microplate reader (Biotek, Winooski, VT, USA).

### Detection of intracellular reactive oxygen species (ROS)

Intracellular ROS production was detected by using the ROS assay kit (Beyotime Company, Shanghai, China) according to the manufacturer’s instructions. Briefly, cells were seeded in a six-well plate at a density of 1 × 10^5^ cells per well. After 24 h incubation under a humidified atmosphere of 5% CO_2_ at 37 °C, the cells were divided into four groups according to the samples added: control group, free Ly (0.5 µg/mL) group, GNS@MSNs-FA/ group (100 µg/mL), and GNS@MSNs-FA/Ly group (100 µg/mL, equivalent Ly dosage of 0.5 µg/mL) was added to each well. Cells without treatment were used as control. After 4 h incubation, GNS@MSNs-FA group and GNS@MSNs-FA/Ly group were treated with 808 nm NIR laser for 5 min at 1.5 W cm^−2^. After cultured for another 20 h, the cells were washed twice with PBS, incubated with DCFH-DA reagent (10 µM) in medium without FBS at 37 °C for 30 min, and then washed with PBS three times. The fluorescence intensity of the cells was observed using a fluorescence microscope (Olympus Corporation, Tokyo, Japan).

### Cell apoptosis

In brief, the cells were seeded in a six-well plate (1 × 10^5^ cells/well) and cultured at 37 ºC in a humidified 5% CO_2_ atmosphere. After culturing for 24 h, the cells were treated according to the description aforementioned. At the end of incubation, all cells were trypsinized, harvested and washed twice with PBS. Then the cells were stained with Annexin V- fluorescein isothiocyanate (FITC) / propidium iodide (PI) dual staining (Nanjing Keygen Biotech, Nanjing, China) according to the manufacturer’s protocol. After incubation in the dark for 20 min at room temperature, the cells were examined by flow cytometry (Becton Dickinson, Franklin Lakes, New Jersey, USA).

### Measurement of ATP levels

The intracellular ATP levels were assessed using an ATP determination kit (Beyotime, China). Briefly, after 24 h of treatment, the cells were harvested and lysed with lysis buffer on ice. After centrifugation for 5 min at 12,000 rpm at 4 °C, the supernatant was collected. After generating a standard curve according to the manufacturer’s protocol, the ATP concentration of the sample was calculated.

### Assessment of intracellular GSH

GSH Assay Kit (Beyotime, China) was used to measure intracellular GSH. In Brief, MNNG/HOS cells were seeded in a six-well plate (1 × 10^5^ cells/well) and cultured overnight. After that, the cells were treated as mentioned above. Subsequently, the treated cells were carefully washed three times with PBS and centrifuged with cell lysate to collect the supernatants. The absorbance of samples was detected at 412 nm by a microplate reader and the contents of GSH in the samples were calculated based on a standard curve.

### Determination of intracellular Ca^2+^ ions

The calcium probe Fluo-3/AM (Dojindo Laboratories Co., Ltd., Kumamoto, Japan) was used to detect the changes of intracellular Ca^2+^ ions according to the manufacturer’s protocol. Briefly, cells were harvested after various treatment and then incubated with 5 μM Fluo-3/AM for 30 min at 37 °C. Then, the cells were carefully washed with PBS to remove excess Fluo-3/AM and the fluorescence intensity was detected by using a flow cytometer (Becton Dickinson, Franklin Lakes, New Jersey, USA).

### Western blotting analysis

Briefly, the treated cells lysed in RIPA buffer (Thermo Fisher Scientific) containing protease inhibitors and phosphatase inhibitors. Subsequently, the lysates were then centrifuged at 12,000 rpm for 15 min at 4 °C and the supernatant was carefully collected. The protein concentrations were measured by using the BCA Protein Assay Kit (Beyotime Biotechnology Co. Ltd). Equal amounts of lysates (20 µg) were electrophoresed by sodium dodecyl-polyacrylamide gel electrophoresis (SDS-PAGE) and transferred onto polyvinylidene difluoride (PVDF) membranes (Millipore, Billerica, MA). The membranes were then blocked with 5% non-fat milk in Tris-buffered saline plus Tween-20 (TBST) buffer for 1 h at room temperature and incubated with the respective primary antibodies at 4 °C overnight. All primary antibodies were used 1:1000 dilution for experiment except GAPDH (1:5,000 dilution). The membranes were then washed with TBST buffer and incubated with peroxidase-conjugated goat anti-rabbit/mouse IgG (Boster no. BA1056 Wuhan, China, 1:10,000 dilution) for 2 h. Subsequently, the membranes were washed three times with TBST buffer and detected by using electrochemiluminescence detection reagent (EMD Millipore) according to the manufacturer’s instructions.

### In vivo imaging and biodistribution analysis

All animal procedures were approved by the Institutional Animal Care and Use Committee (IACUC) at Tongji Medical College, Huazhong University of Science and Technology (IACUC Number: S2374). In brief, a volume of 200 μL MNNG/HOS cell suspension (density of 1 × 10^7^ cells/mL) in cold PBS was subcutaneously injected into the right flank of nude mice.

When the volume of tumors reached 60–100 mm^3^, the mice were randomly divided into two groups (n = 3): (1) GNS@MSNs and (2) GNS@MSNs-FA. 150 μL ICG labeled NPs (2 mg mL^−1^) were intravenously injected through the tail vein into the mice bearing the tumor. At the given time intervals (0, 1, 6 and 24 h), in vivo tumor imaging was obtained by IVIS small animal imaging system (PerkinElmer Inc., Waltham, USA). After 24 h of observation, the mice were sacrificed and main organs (heart, lung, liver, spleen, and kidney) as well as tumors were harvested for ex vivo imaging to study the tissue distribution of NPs.

### In vivo antitumor efficacy and biosafety

When the tumor could be palpated subcutaneously (5 days after inoculation), the mice were randomly allocated into seven groups: (1) PBS, (2) NIR, (3) free Ly, (4) free Ly + NIR, (5) GNS@MSNs-FA/Ly, (6) GNS@MSNs-FA + NIR, and (7) GNS@MSNs-FA/Ly + NIR (n = 5 per group). 100 μL free Ly (10 mg kg^−1^) was injected intraperitoneally every three days. Other therapeutic agents were intravenously injected into the mice via the tail vein every three days. After 6 h of injection, the NIR laser treatment groups were irradiated with an 808 nm laser for 5 min (1.0 W cm^−2^).

The body weights and tumor sizes were measured every 2 days to observe the growth of the tumors. At the end of the experiment (12 days after treatment), all mice were sacrificed. Tumor tissues as well as major organs (heart, liver, spleen, lung, and kidney) of mice were collected and fixed with 4% paraformaldehyde and embedded in paraffin blocks, and then sectioned into 5-μm sections. After stained with hematoxylin and eosin (H&E), the sections were observed using an optical microscope for histological observation and morphometric analysis. In addition, the tumors slides were used for immunofluorescence-stain with Ki67 antibody and terminal deoxynucleotidyl transferase-mediated dUTP nick end labeling (TUNEL) staining. Images of sections were obtained using a light microscope.

### Blood biochemical assay

To further affirm the biosafety of all treatment, the liver or kidney function was evaluated by determining the serum level of alanine aminotransferase (ALT) or blood urea nitrogen (BUN). Briefly, the blood from mice were collected and centrifuged at 8000 rpm for 5 min to obtain the serum. Subsequently, the levels of ALT and BUN in serum samples were measured by hematology analyzer.

### Statistical analysis

All data were expressed as mean ± standard deviation (SD) from at least three independent experiments under the same experimental conditions. Statistical analysis was conducted with Student’s t-test and one-way analysis of variance (ANOVA) by using GraphPad Prism version 6.01 for Windows. A *P*-value < 0.05 was considered to show statistically significant differences.

## Results and discussion

### Synthesis and characterization of GNS@MSNs-FA/Ly

**Fig. 1:**
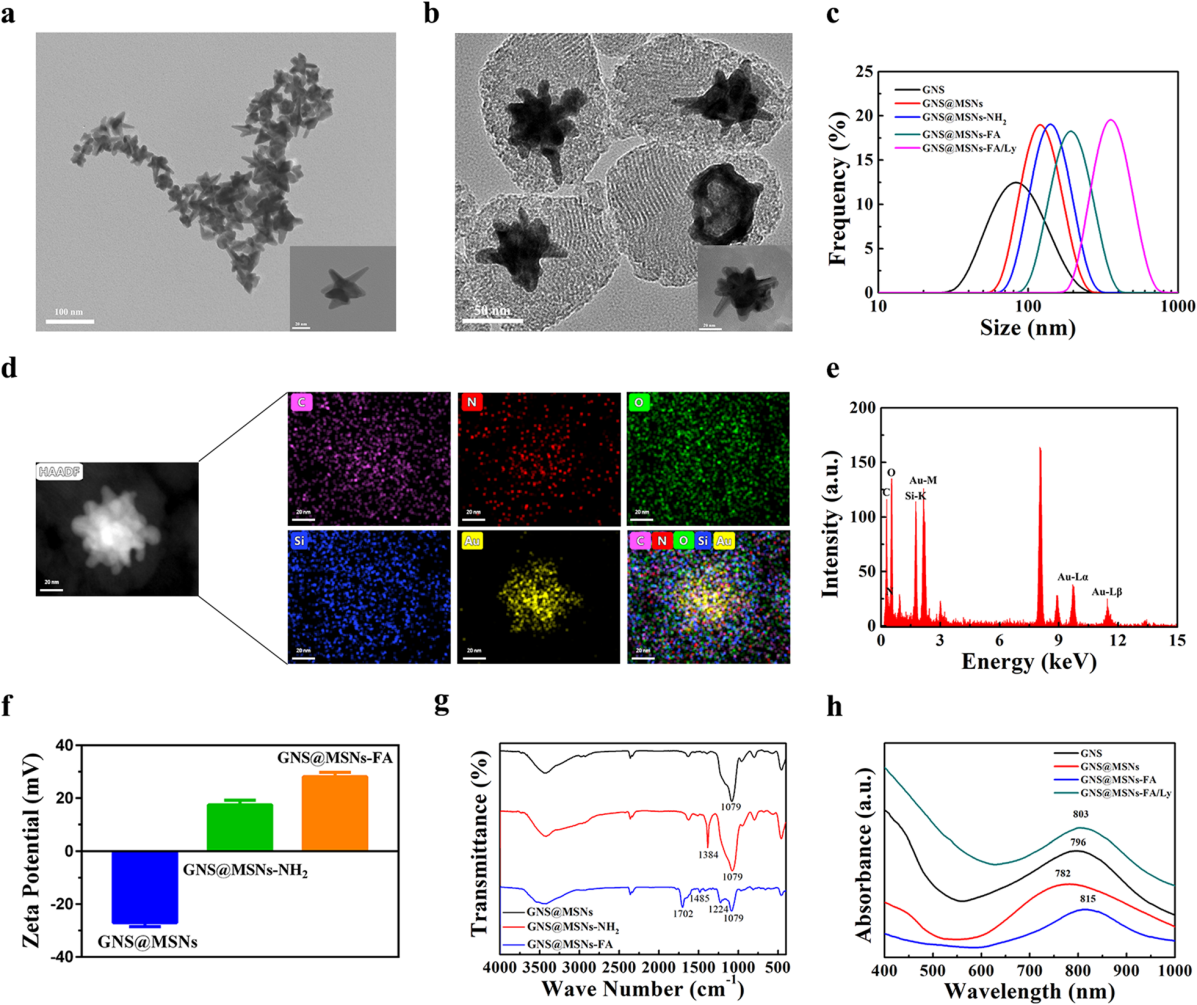
Preparation and characterization of GNS@MSNs-FA and GNS@MSNs-FA/Ly. **a**, **b** TEM images of GNS and GNS@MSNs-FA. **c** The DLS size of GNS, GNS@MSNs, GNS@MSNs-NH2, GSN@MSNs-FA, and GSN@MSNs-FA/Ly. **d**, **e** EDX mapping and analysis of the GNS@MSN-FA. **f** Zeta potential of GNS@MSNs, GNS@MSNs-NH_2_, and GNS@MSNs-FA. **g** FTIR spectra of GNS@MSNs, GNS@MSNs-NH_2_, and GNS@MSNs-FA. **h** UV–vis absorption spectra of GNS, GNS@MSNs, GNS@MSNs-FA, and GSN@MSNs-FA/Ly

The detailed design of GNS@MSNs-FA/Ly was displayed in Scheme 1a. Firstly, GNSs were synthesized via a seed-mediated growth method with minor modification [42, 43]. Transmission electron microscope (TEM) image (Fig. 1a) informed that the resultant GNSs contained many "spiny" structures and the diameter ranged from 50 to 80 nm. A mesoporous silica shells were coated on the surface of GNSs by a Modified Stöber method. The obtained GNS@MSNs-FA NPs were observed by TEM which demonstrated particles size around 120 nm and a core–shell structure with a well-defined porous structure, providing a high capacity for drug loading (Fig. 1b). The particle size distribution was measured by dynamic light scattering (DLS) analysis. As shown in Fig. 1c, the hydrodynamic diameter of GNS, GNS@MSNs, GNS@MSNs-NH_2_, GNS@MSNs-FA, and GNS@MSNs-FA/Ly were 53.8 nm (PDI = 0.361), 122.0 nm (PDI = 0.235), 156.1 nm (PDI = 0.288), 213.6 nm (PDI = 0.344), and 363.7 nm (PDI = 0.31), respectively. Of note, the size obtained from DLS was larger than that measured by TEM, possibly because DLS result showed a hydrodynamic particle size whereas TEM image displayed a dehydration morphology [47]. Moreover, the hydrodynamic size variation of the GNS@MSNs-FA/Ly NPs were monitored in water, α-MEM culture medium with or without 10% FBS over a prolonged incubation time up to 7 days. As shown in the revised Additional file 1: Fig S1, no notable size changes were found as time went on. These characterizations demonstrated the good stability of the NPs in physiological solution, which would benefit their long-term circulation in the body. Additionally, the elemental mapping images (Fig. 1d) clearly presented that the Au element from GNS was located in the core, whereas the Si and O element from mesoporous silica were on the outside shell, which further confirms the well-defined core–shell structure of the GNS@MSNs. Moreover, the presence of C and N signals derived from FA, which further confirmed the successful modification of FA. EDX analysis also verified the elemental components in the bioconjugate (Fig. 1e).

The modification process of the GNS@MSNs was assessed by zeta potential and FTIR. As shown in Fig. 1f, the zeta potentials of GNS@MSNs, GNS@MSNs-NH_2_, and GNS@MSNs-NH_2_-FA were − 27.1 ± 1.5, 17.5 ± 1.7 and 28.1 ± 1.6 mV, respectively. This inverted and fluctuated zeta potential changes provided compelling evidence on the successful functionalization of GNS@MSNs in each step. Moreover, FTIR spectroscopy was performed to monitor the surface chemistry changes along the modification process. As demonstrated in Fig. 1g, the FTIR spectra of GNS@MSNs, GNS@MSNs-NH_2_ and GNS@MSNs-FA all exhibited the characteristic Si–O-Si vibration peak at 1079 cm^−1^, confirming the coating of the silica shell. The newly emerged typical bonds at 1384 cm^−1^ (C-H bending) in GNS@MSNs-NH_2_ proved the successful formation of an amide bond. After modification by FA, the shoulder peaks appeared at 1224 and 1485 cm^−1^ in GNS@MANs-FA, ascribing to the stretching mode of C=C and C-O in the pteridine ring, respectively. In addition, the characteristic peaks at 1702 cm^−1^ for GNS@MSNs-FA can be indexed to the stretching mode of C=O.

Subsequently, the LSPR spectrum of GNS, GNS@MSNs, GNS@MSNs-FA, and GNS@MSN-FA/Ly were characterized. As displayed in Fig. 1h, the typical LSPR peak of as-prepared GNS was at 796 nm. After coating mesoporous silica shell, the red shift of LSRP peak appeared due to the change of local refractive index [48]. Finally, the modification of FA made the LSPR peak further shifted to 815 nm and GNS@MSN-FA/Ly has a LSPR peak at 803 nm, which located in the NIR region and displayed remarkable potential for light-mediated therapy.

### Photothermal properties of GNS@MSNs-FA

**Fig. 2:**
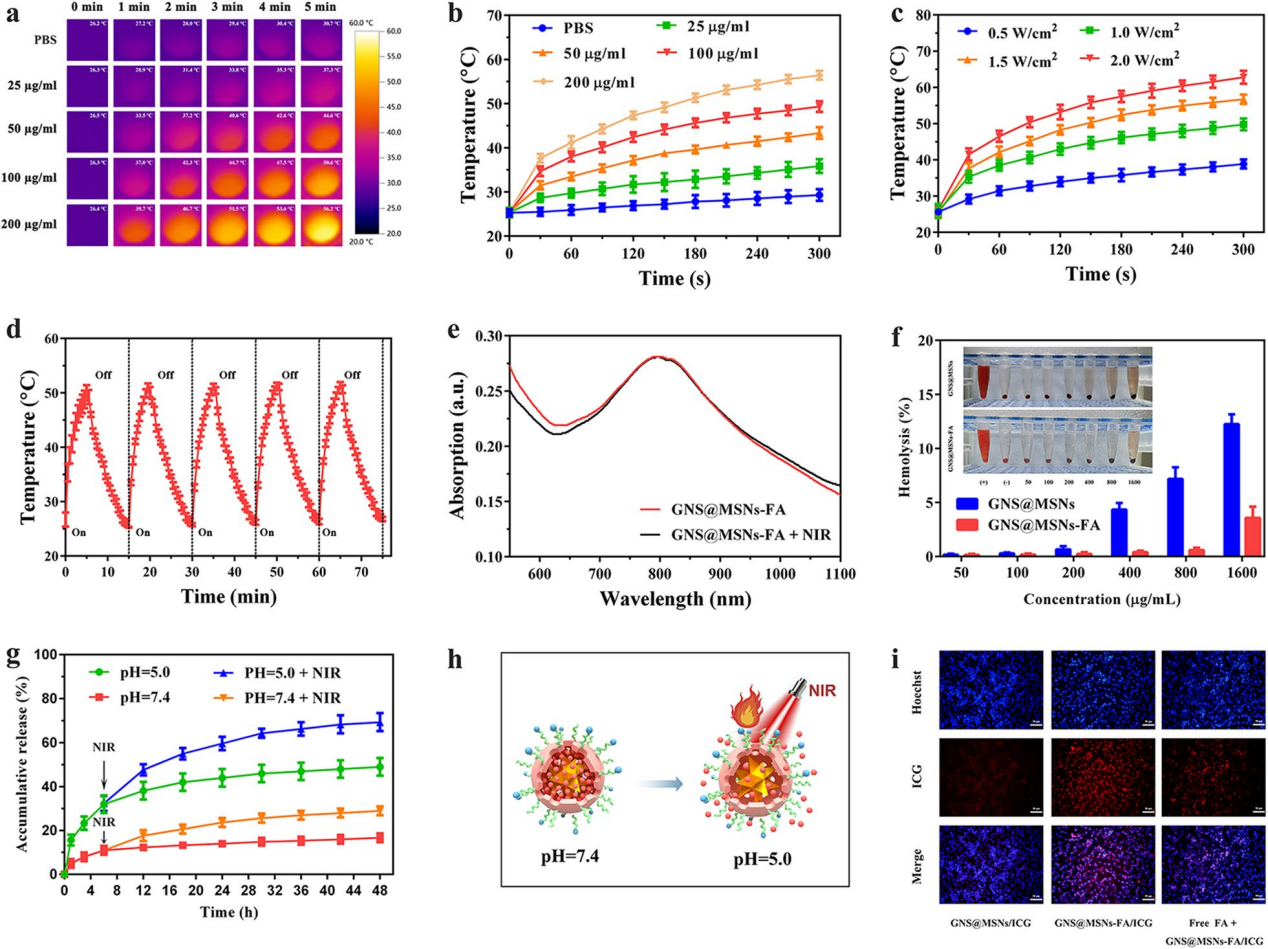
Properties of GNS@MSNs-FA. **a**, **b** The infrared thermal images and time-dependent temperature changes of GNS@MSNs-FA dispersion with different concentrations under 1 W cm^−2^ 808 nm laser irradiation. **c** Time-dependent temperature change of GNS@MSNs-FA (100 µg mL^−1^) under 808 nm laser irradiation with different laser power. **d** Photothermal stability of GNS@MSNs-FA under 808 nm laser irradiation (1 W cm^−2^). **e** UV–vis spectra of GNS@MSNs-FA (100 μg mL^−1^) dispersed in PBS before and after 1 h of NIR laser irradiation. **f** Hemolysis assay of GNS@MSNs and GNS@MSNs-FA samples. **g** Cumulative release of Ly from GNS@MSNs-FA/Ly nanocomposites in PBS with different pH values without or with NIR irradiation (808 nm, 1.0 W cm^−2^, 5 min). **h** Illustration of the pH and NIR-laser-triggered release behavior of GNS@MSNs-FA. **i** Fluorescence microscopy images of MNNG/HOS cells after incubation with GNS@MSNs/ICG, GNS@MSNs-FA/ICG and GNS@MSNs-FA/ICG + free FA for 4 h

In order to evaluate the photothermal properties of GNS@MSNs-FA in vitro, the temperature changes of NPs after NIR irradiation (808 nm) were detected in real-time by an infrared thermal imaging camera. As shown in Fig. 2a and b, compared with the slight temperature variation of the PBS, the temperature of GNS@MSNs-FA significantly increased in a concentration- and time-dependent manner. Besides, a significant laser power intensity-dependent manner could be also observed (Fig. 2c). The excellent photothermal conversion efficiency might be attributed to the LSPR of GNS [49]. Furthermore, the photostability of GNS@MSNs-FA upon the NIR irradiation at 1.0 W cm^−2^ for 5 min was assessed. After five cycles of irradiation on/off with an 808 nm laser, the temperature variation curves and peak shape were of no obvious change (Fig. 2d). Besides, the UV–vis absorbance spectra of GNS@MSNs-FA was almost congruent (Fig. 2e) before and after 2 h of NIR irradiation, suggesting the promising photothermal stability of GNS@MSNs-FA. All these results taken together indicated that the excellent photothermal conversion property and outstanding photothermal stability made GNS@MSNs-FA good candidates in the PTT of cancers.

### Biocompatibility of NPs

The biocompatibility of NPs is of great significance for their systemic administration as drug delivery carriers [37]. Hemolysis is the crucial risk project related to the biocompatibility of NPs intended for injection [50]. The hemolysis assay was performed to assess the hemocompatibility of GNS@MSNs-FA. It was found in Fig. 2f that the hemolysis percentages of GNS@MSNs did not change obviously until the concentration reached 400 μg mL^−1^. However, the hemolysis percentages of GNS@MSNs-FA were less than 3% even at the highest concentration of 1600 μg mL^−1^. Moreover, the cytotoxicity of GNS@MSNs-FA was evaluated by CCK8 assay. Normal human cells BMSCs were selected for the cytotoxicity testing. As displayed in Additional file 1: Fig. S2, GNS@MSNs-FA exhibited no significant cellular toxicity to the cell lines after 24 h treatment, even at a concentration of NPs up to 800 µg/mL, indicating low cytotoxicity of GNS@MSNs-FA. All these findings revealed that the as-synthesized GNS@MSNs-FA is an extraordinarily biocompatible nanoplatform, which encourages us to utilize the nanocomposites as drug delivery carriers.

### Drug loading and release

The poor aqueous solubility and insufficient target specificity restricted the clinical applications of Ly. Therefore, MSN-based multifunctional nanocarriers (GNS@MSNs-FA) were developed and used as reservoirs for delivering Ly. From the UV–vis spectra results in Additional file 1: Fig. S3, the as-synthesized GNS@MSNs-FA/Ly showed a typical absorption peak at 291 nm, which can also be found in the spectra of free Ly, indicating that Ly was successfully loaded into GNS@MSNs-FA. Excitingly, according to the absorption spectra and standard curve of Ly (Additional file 1: Fig. S4a and b), the drug loading degree was about 37.6 ± 3.3% and the corresponding encapsulation efficiency was about 30.2 ± 4.2%.

**Fig. 3:**
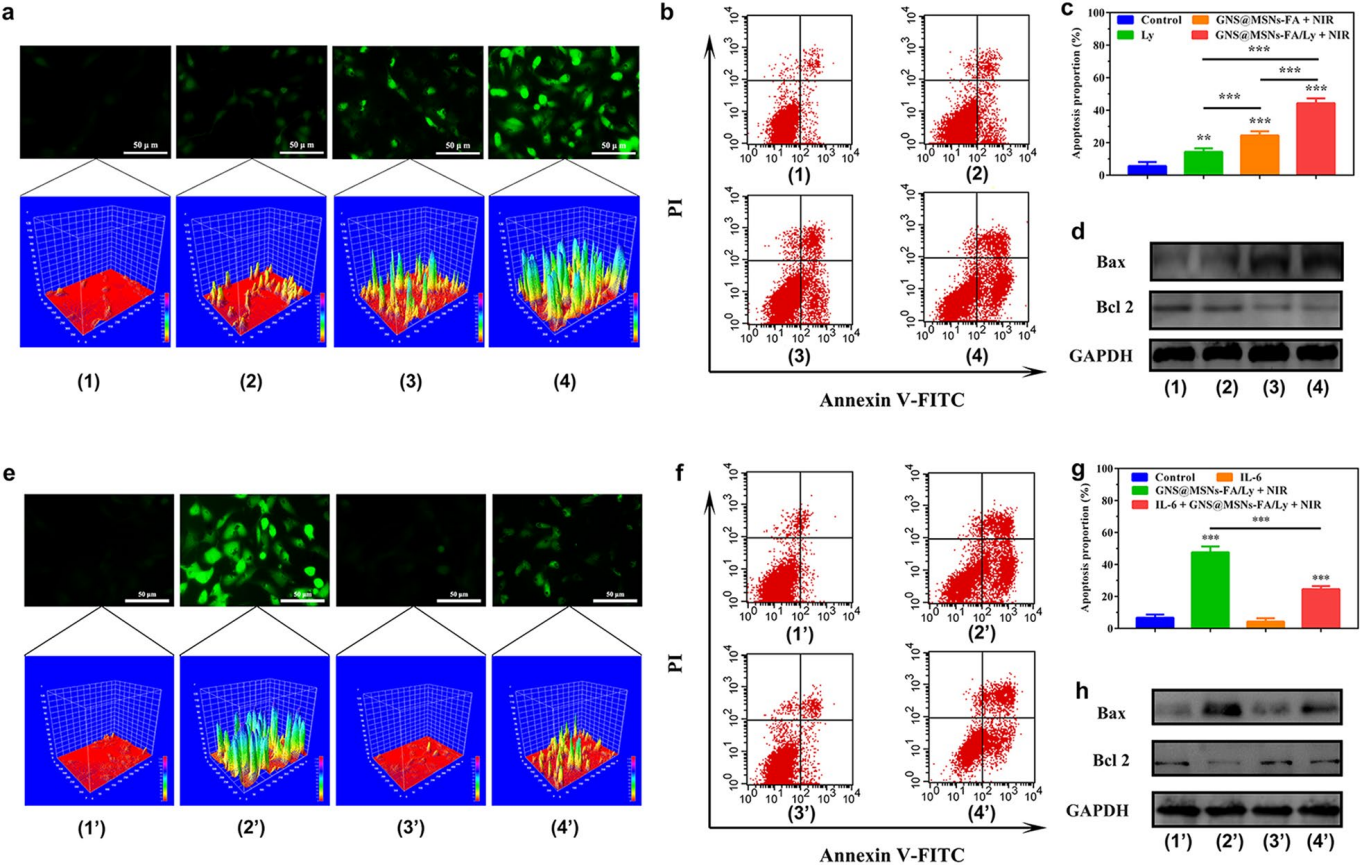
The ROS overproduction contributed to the synergistic anti-tumor effect. **a** Fluorescence microscopy and corresponding surface plot images of ROS generation under the presence of DCFH-DA in MNNG/HOS cells after corresponding treatment for 24 h. **b**, **c** MNNG/HOS cells after corresponding treatment for 24 h were stained with Annexin V-FITC/PI and analyzed by flow cytometry. **d** The expression of Bax and Bcl-2 in MNNG/HOS cells after indicated treatment (Groups included (1) control, (2) Ly, (3) GNS@MSNs-FA + NIR, (4) GNS@MSNs-FA/Ly + NIR). **e** The ROS scavenger NAC partially attenuated GNS@MSN-FA/Ly coupled with NIR -induced ROS accumulation in MNNG/HOS cells. **f**, **g** NAC partially attenuated the pro-apoptotic effect of GNS@MSNFA/Ly coupled with NIR on MNNG/HOS cells. **h** NAC partially abolished the effect of GNS@MSNFA/Ly coupled with NIR on the expression of Bax and Bcl-2 in MNNG/HOS cells (Groups included (1’) control, (2’) GNS@MSNs-FA/Ly + NIR, (3’) NAC, (4’) NAC + GNS@MSNs-FA/Ly + NIR)**.** (*P < 0.05, **P < 0.01, ***P < 0.001)

Considering the slightly acidic pH of endo/lysosomes in cancer cells, we next investigated the release behavior of Ly from GNS@MSNs-FA/Ly under different pH conditions. As exhibited in Fig. 3d, less than 20% of Ly was released in neutral PBS buffer (pH 7.4), but it was found that the accumulative release of Ly in pH 5.0 increased up to 40% after 48 h of incubation. The results demonstrated that the Ly release form GNS@MSNs-FA/Ly was pH-dependent. Therefore, it can be expected that more Ly will be easily released from the nanocomposites under acidic tumor microenvironment [51]. In addition, the NIR-laser-triggered release behavior of GNS@MSNs-FA/Ly was carefully investigated at pH 7.4 and pH 5.0 as well. As illustrated in Fig. 2g, the drug release rates were slightly increased at pH = 7.4 with NIR-laser exposure. Excitingly, upon laser irradiation, an obvious burst release of Ly from GNS@MSNs-FA/Ly can be observed at pH 5.0. The accelerated drug release might be ascribed to the improved photothermal conversion efficiency of GNS at the NIR region of the electromagnetic spectrum, which could increase the local temperature [52]. With the increase of local temperature, molecular desorption is triggered by the thermal movement of the crystalline lattice of the carrier, and the mobility of temperature-dependent drug molecules is accelerated, which leads to the accelerated desorption and release of Ly from GNS@MSNs-FA/Ly [30, 37]. The above results revealed that the nanocomposites could respond to intrinsic low pH of endo/lysosomes in cancer cells and extrinsic flexible NIR to programmed release loaded Ly could exhibit dual pH/thermal-responsive Ly release property (Fig. 2h). Such pH and NIR-laser-triggered release behavior will be useful for intelligently controlled drug release and beneficial for synergistic cancer therapy.

### Cellular uptake

The internalization of nanocomposites into targeted cells is of paramount importance for nanoparticulate drug delivery systems [53]. A common strategy for enhancing the internalization of NPs in cancer cells is to decorate the surface of NPS with targeted ligands [54]. The FOLR has been reported to be overexpressed in various cancer cells, including OS cells [39, 40]. In this study, FA molecules were conjugated on the surface of GNS@MSNs-NH_2_ hoping to enhance the intracellular accumulation. Of note, ICG was used as a fluorescent agent. As illustrated in Fig. 2i and Additional file 1: Fig. S5, the GNS@MSNs-FA/ICG nanohybrids displayed higher red fluorescence signals in cell cytoplasm after 4 h of incubation as compared to GNS@MSNs/ICG. Furthermore, it was obvious from the FA molecule competing assay that the uptake of GNS@MSNs-FA/ICG was significantly reduced by the pre-treated free FA molecules. The results strongly suggested that the modification of FA on the surface of GNS@MSNs could enhance the intracellular accumulation.

### The ROS overproduction contributed to the synergistic anti-tumor effect

To assess the intracellular ROS production, the DCFH-DA fluorescent probe staining assay was firstly conducted. As shown in Fig. 3a, the GNS@MSNs-FA/Ly + NIR group exhibited higher intracellular ROS levels than that of free Ly and GNS@MSNs-FA + NIR group under the same conditions. It is known that excessive ROS production might lead to cancer cell death via imbalance of redox homeostasis [18]. To assess the synergistic anti-tumor effect, the cell viability was analyzed using the CCK8 assay. As displayed in Additional file 1: Fig. S6, GNS@MSNs-FA/Ly in the presence of NIR laser irradiation (5 min, 1.0 W cm^−2^) displayed highest cytotoxicity to MNNG/HOS cells. Only about 38% cells kept alive after being treated with GNS@MSNs-FA/Ly + NIR for 24 h, while there were 71% or 60% of viable cells for those treated with Ly or GNS@MSNs-FA + NIR, respectively. Consistently, we found that GNS@MSNs-FA/Ly + NIR could induce a much higher level of cell apoptosis compared to Ly or GNS@MSNs-FA + NIR (Fig. 3b, c). Furthermore, the levels of apoptosis‐related proteins were also investigated. It was found that the combined treatment group exhibited the highest level of proapoptotic protein Bax, while significantly reduced the anti-apoptosis protein Bcl-2 (Fig. 3d and Additional file 1: Fig. S7).

N-Acetyl-L-cysteine (NAC), a classic ROS scavenger, was used to verify whether the ROS generation contributed to the synergistic anti-tumor effect. As shown in Fig. 3e, pretreatment of NAC could partially abrogate the ROS production induced by the combined therapy. Furthermore, the pro-apoptotic effect of the combined therapy could be partially rescued (Fig. 3f, g) and its effect on protein expression (Bax and Bcl-2) was also partially abolished (Fig. 3h and Additional file 1: Fig. S8). Collectively, all these results confirmed that GNS@MSNs-FA/Ly coupled with NIR irradiation could offer excellent cell growth inhibition effect via the apoptosis pathway due to the amplified intracellular oxidative stress.

### Amplification of oxidative stress by inducing mitochondrial dysfunction and potent ER stress

**Fig. 4:**
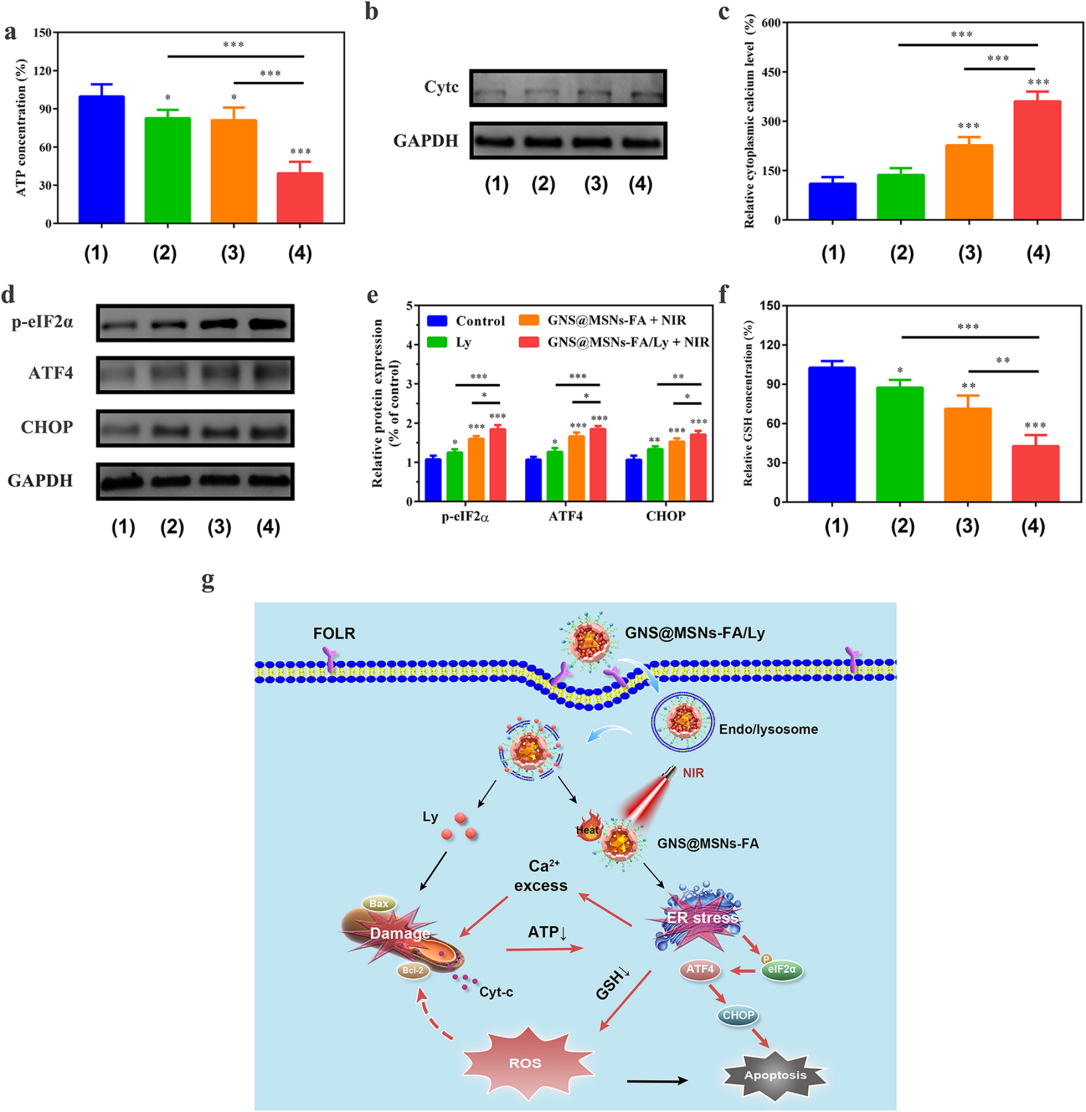
Amplification of oxidative stress by inducing mitochondrial dysfunction and potent ER stress. **a** Intracellular ATP levels of MNNG/HOS cells after corresponding treatment. **b** The expression of Cyt-c in MNNG/HOS cells after indicated treatment. **c** Relative cytoplasmic calcium level in in MNNG/HOS cells after indicated treatment. **d**, **e** The effect of various treatment on the expression of ER stress-related proteins. **f** Relative GSH concentration of MNNG/HOS cells after corresponding treatment. (Groups included (1) control, (2) Ly, (3) GNS@MSNs-FA + NIR, (4) GNS@MSNs-FA/Ly + NIR). **g** A schematic illustration of the underlying mechanism for the synergistic cascade cancer therapy. (*P < 0.05, **P < 0.01, ***P < 0.001)

As fundamental organelles in cell metabolism, mitochondria are responsible for ROS production, ATP synthesis, and cell death [17, 55]. Mitochondria dysfunction may impair the electron transport chain, leading to decreased ATP synthesis and increased ROS generation. When ROS levels produced by mitochondria exceed the antioxidant defenses, oxidative stress is generated [55]. It is well known that mitochondrial dysfunction is usually accompanied by ATP depletion and Cyt-c release from the mitochondria into the cytosol [56]. To determine whether the synergistic anti-tumor effect was associated with mitochondrial dysfunction, the intracellular ATP levels and Cyt-c release after various treatment were detected. As shown in Fig. 4a, the intracellular ATP levels were found to be dramatically decreased in cells treated with GNS@MSNs-FA/Ly + NIR, to the level much lower than that observed in cells treated with Ly or GNS@MSNs-FA + NIR alone. Moreover, the Western blot analysis also demonstrated that GNS@MSNs-FA/Ly + NIR significantly promoted Cyt-c release from the mitochondria into the cytosol (Fig. 4b and Additional file 1: Fig. S9). Previous studies showed that ER stress can be induced by ATP deprivation and gold-based NPs [23, 57, 58]. In ER-stressed cells, Ca^2 +^ released from the ER is taken up by mitochondria and subsequently induces mitochondrial Ca^2+^ overload, eventually leading to mitochondrial oxidative stress and dysfunction [17, 22]. Therefore, the cytosolic calcium levels, a known indicator of ER stress, were determined by using the calcium-sensitive Fluo-3-AM probe. As shown in Fig. 4c, the levels of cytoplasmic calcium in cells treated with GNS@MSNs-FA/Ly + NIR higher than that of Ly or GNS@MSNs-FA + NIR. Consistently, the levels of ER stress-related proteins, including p-eIF-2α, ATF4 and CHOP, were all significantly upregulated in cells treated with GNS@MSNs-FA/Ly + NIR (Fig. 4d). More importantly, the occurrence of ER stress might lead to depleting ER GSH levels (Fig. 4e, f). As predicted, we found that GNS@MSNs-FA + NIR could lead to a significant decrease in intracellular GSH. Taken together, all these results indicated that GNS@MSNs-FA/Ly + NIR could significantly amplify the intracellular oxidative stress by causing more severe mitochondrial dysfunction and more powerful ER stress (Fig. 4g).

### Biodistribution and in vivo photothermal effect

**Fig. 5:**
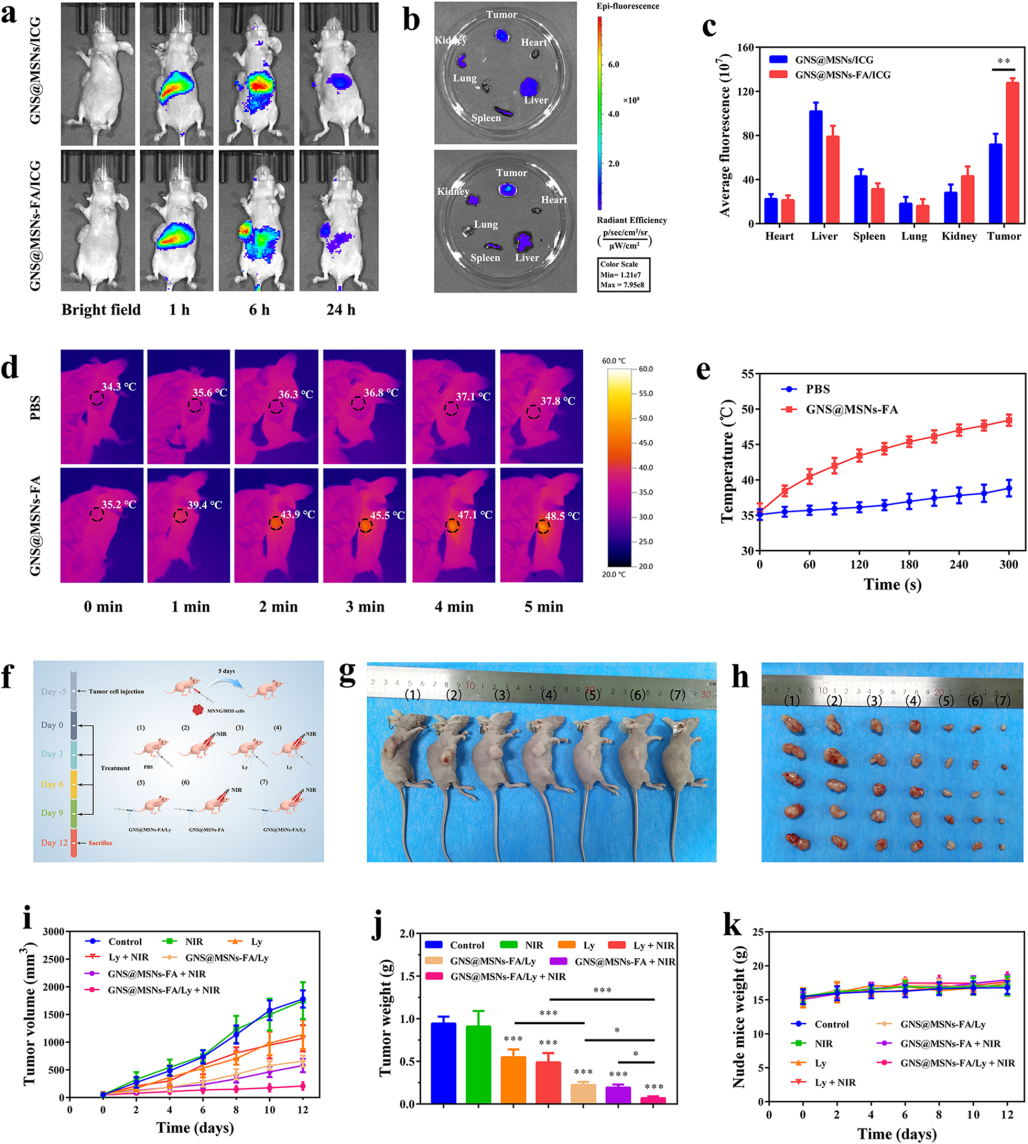
In vivo synergistic combined therapy of OS with GNS@MSNs-FA/Ly coupled with NIR irradiation. **a** In vivo fluorescence images of MNNG/HOS tumor-bearing mice at indicated time point following iv injection with ICG labeled NPs (GNS@MSNs and GNS@MSNs-FA). **b** Ex vivo fluorescence images of the main organs and tumor at 24 h post-injection. **c** Semiquantitative analysis of ICG fluorescence intensity around the tumors 24 h post-injection. **d**, **e** In vivo thermal images and temperature rising curves of MNNG/HOS tumor-bearing mice at 6 h post-injection of PBS and GNS@MSNs-FA with an 808 nm laser-irradiation (1.0 W cm^−2^). **f** Schematic diagram of in vivo therapy in tumor-bearing mice. **g** Representative photographs of tumor-bearing mice after 12 d of various treatments. **h** The photographs of all tumors taken from mice after 12 d of different treatments. (Groups included (1) control, (2) NIR, (3) Ly, (4) Ly + NIR, (5) GNS@MSNs-FA/Ly, (6) GNS@MSNs-FA + NIR, (4) GNS@MSNs-FA/Ly + NIR). **i** Tumor volume growth curves of tumor-bearing mice during the therapies with various formulations. **j** Tumor weights were measured after 12 days of various treatments. **k** Body weight changes of MNNG/HOS tumor-bearing mice in different groups. (* P < 0.05, ** P < 0.01, *** P < 0.001)

To examine the in vivo tumor-targeting ability and distribution of NPs, MNNG/HOS tumor-bearing mouse model was established for in vivo imaging by using an IVIS small animal imaging system. As shown in Fig. 5a, the fluorescence signals in the tumors gradually increased over time, indicating the accumulation process of NPs (GNS@MSNs/ICG and GNS@MSNs-FA/ICG) in the tumors. In comparison with the GNS@MSNs/ICG treatment group, the tumor site in the GNS@MSNs-FA/ICG treatment group showed stronger fluorescence at 6 h and 24 h, indicating the FA modification might improve the tumor targeting of NPs. After 24 h administration, the mice were sacrificed, and the tumor tissues as well as major organs (heart, liver spleen, lung and kidney) were harvested for ex vivo imaging. Obviously, the fluorescence intensity of tumor tissue in GNS@MSNs-FA/ICG treatment group was still much stronger than that in GNS@MSNs/ICG treatment group (Fig. 5b, c), further confirming the superior enrichment capacity of GNS@MSNs-FA in tumor. Taken together, the efficient tumor accumulation effect of GNS@MSNs-FA/ICG NPs might be contributed to the enhanced permeability and retention (EPR) effect-mediated passive and FA-induced active delivery [59–61].

Based on the above in vivo imaging observation, 6 h post-injection was selected as the optimal time point for laser irradiation. Thus, we further evaluated the laser-triggered photothermal effect of GNS@MSNs-FA in vivo 6 h after intravenous administration of the NPs. As displayed in the photothermal images (Fig. 5d) and time–temperature curves (Fig. 5e), the local tumor temperature could increase over 15 °C after irradiation at 1.0 W cm^−2^ for 5 min, which was sufficient to ablate malignant cells irreversibly [34, 62]. In marked contrast, in the PBS group, the same irradiation only resulted in a little temperature increase of the tumor. These results highlighted the promising photothermal performance of GNS@MSNs-FA in vivo.

### In vivo antitumor therapy

Encouraged by the effective accumulation of GNS@MSNs-FA in tumor and the satisfying cytotoxicity results in vitro, we further evaluated the in vivo antitumor effect of GNS@MSNs-FA/Ly in MNNG/HOS tumor-bearing mice. The experimental process in vivo was displayed in Fig. 5f. As shown in Fig. 5 g–j, no obvious tumor growth inhibition was observed in NIR laser irradiation treatment group compared with the control, demonstrating that NIR irradiation alone was nearly no effect on suppressing the tumor growth. Unsurprisingly, the more sufficient growth suppression of tumor was observed on the mice injected with GNS@MSNs-FA/Ly coupled with NIR laser irradiation, which verified the excellent synergistic therapeutic effect. Notably, no significant weight loss was observed in all groups during the treatment period (Fig. 5k).

**Fig. 6:**
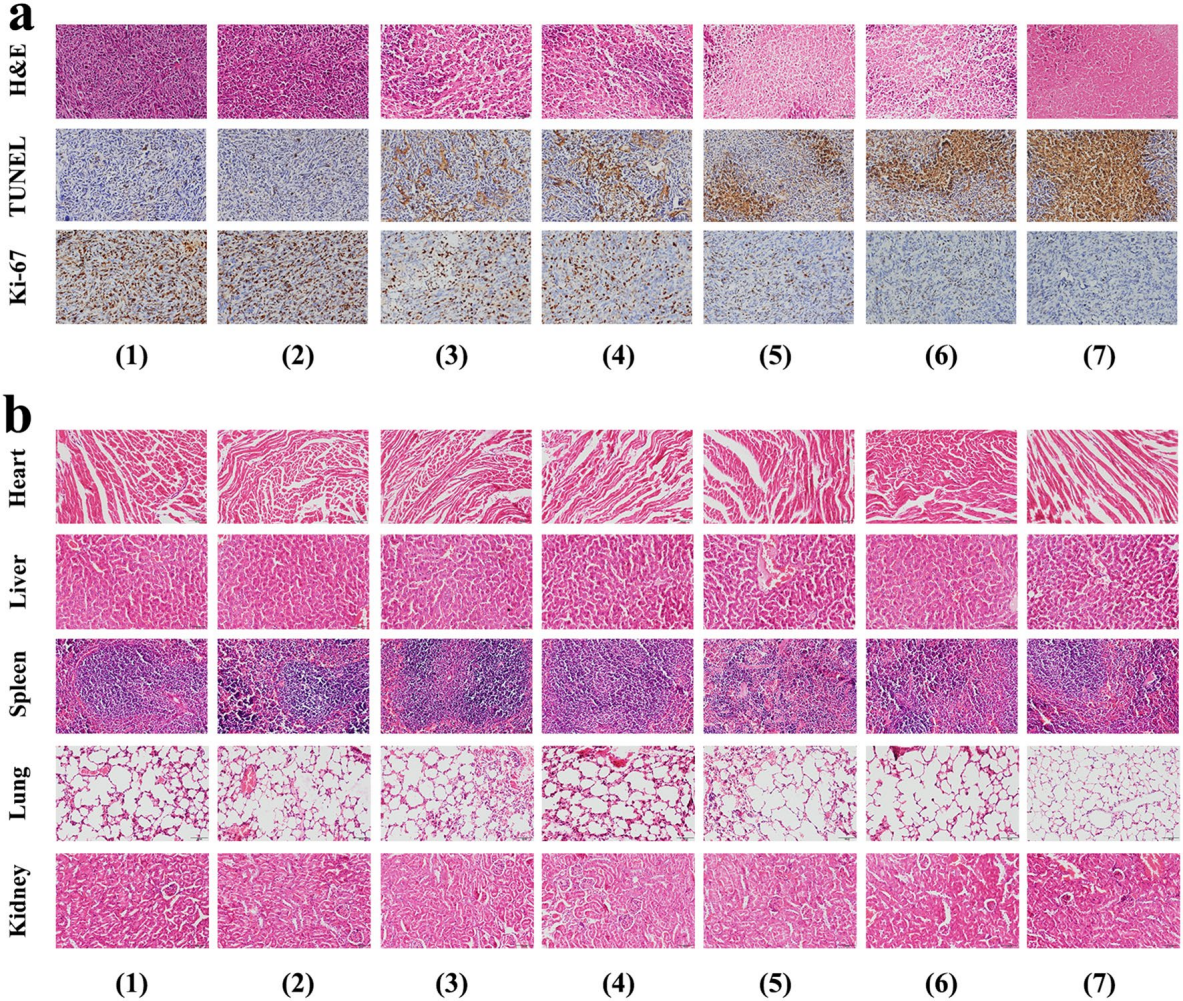
Histological analyses.** a** H&E and immunohistochemical staining with TUNEL and Ki-67 analyses of tumor slices in different groups. **b** H&E-stained images of major organs of MNNG-HOS bearing mice after the various treatments. (Groups included (1) control, (2) NIR, (3) Ly, (4) Ly + NIR, (5) GNS@MSNs-FA/Ly, (6) GNS@MSNs-FA + NIR, (4) GNS@MSNs-FA/Ly + NIR)

Moreover, the therapeutic effects of various treatment were further demonstrated by H&E and TUNEL staining of tumor tissues. As exhibited in H&E staining images (Fig. 6a), a more extensive necrosis was found in tumor tissues treated with GNS@MSNs-FA/Ly coupled with NIR irradiation than that of free Ly or GNS@MSNs-FA + NIR group. TUNEL staining also revealed that GNS@MSNs-FA/Ly coupled with NIR irradiation caused cell apoptosis at a higher level compared with other treatments, in accordance with the tumor growth trends. Meanwhile, the expression of proliferative marker Ki‐67 was also evaluated to further confirm the inhibitory role of synergistic therapeutic efficacy on tumor growth. Consistent with tumor sizes and weights, the tumors treated with GNS@MSNs-FA/Ly coupled with NIR irradiation showed lower expression of Ki67 (Fig. 6a). Furthermore, H&E staining of major organs (heart, liver, spleen, lung, and kidney) also confirmed that no obvious tissue damage was observed among all groups (Fig. 6b). Meanwhile, the levels of liver and kidney function markers (ALT and BUN) were all within normal range (Additional file 1: Fig. S10a and b), indicating all treatments had no significant systemic toxicity to the animals. These results collectively certified the favorable biocompatibility and systemic safety of GNS@MSNs-FA/Ly.

## Conclusion

In summary, we have successfully developed a biocompatible nano-formulation, GNS@MSNs-FA/Ly, which is composed of a cargo of TCM herb (Ly), a gold-based NP core (GNS), a mesoporous silica-based shell, conferring efficient cancer combination therapy. GNS@MSNs-FA/Ly couple with NIR irradiation could promote a high level of ROS production via inducing mitochondrial dysfunction and potent ER stress. Moreover, GSH depletion during ER stress could reduce ROS scavenging and further enable efficient amplification of intracellular oxidative stress. Both in vitro and in vivo studies demonstrated that GNS@MSNs-FA/Ly coupled with NIR irradiation exhibited excellent antitumor efficacy with high specificity and no additional side effect. Taken together, this promising strategy offers a new avenue for the effective cancer synergetic therapy and future clinical translation.

## Supplementary Information

**Additional file 1**: **Figure S1**. Hydrodynamic size variation of the GNS@MSNs-FA/Ly NPs dispersed in water, α-MEM culture medium with or without 10% FBS. **Figure S2**. Cell viability of BMSCs cells after 24 h of treatments with various concentrations of GNS@MSNs-FA. **Figure S3**. UV-Vis spectra of Ly, GNS@MSNs-FA and GNS@MSNs-FA/Ly. **Figure S4**. a The absorption spectra of Ly with different concentrations. b The standard curve of Ly determined by a UV-VIS spectrophotometer. **Figure S5**. The corresponding surface plot of ICG fluorescence images of MNNG/HOS cells after incubation with GNS@MSNs/ICG, GNS@MSNs-FA/ICG and GNS@MSNs-FA/ICG + free FA for 4h. **Figure S6**. Cell viability of MNNG/HOS cells after the corresponding treatment for 24 h. **Figure S7**. Relative protein levels of Bax and Bcl-2 in MNNG/HOS after various treatment. **Figure S8**. Relative protein levels of Bax and Bcl-2 in MNNG/HOS after various treatment. **Figure S9**. Relative protein levels of Cyt-c in MNNG/HOS after various treatment. **Figure S10**. Biosafety evaluation by blood biochemistry test. a Serum levels of ALT (liver function index). b Serum levels of BUN (kidney function index).

## Data Availability

The datasets supporting the conclusions of this article are included within the article and its additional file.
