# Supplementary material for: Amplification of oxidative stress with lycorine and gold-based nanocomposites for synergistic cascade cancer therapy
Source: J Nanobiotechnology. 2021 Jul 27;19:221. doi: 10.1186/s12951-021-00933-1 (PMC8314456; doi:10.1186/s12951-021-00933-1)
Supplement: Supplementary file 1 — Additional file 1: Figure S1. Hydrodynamic size variation of the GNS@MSNs-FA/Ly NPs dispersed in water, α-MEM culture medium with or without 10% FBS. Figure S2. Cell viability of BMSCs cells after 24 h of treatments with various concentrations of GNS@MSNs-FA. Figure S3. UV-Vis spectra of Ly, GNS@MSNs-FA and GNS@MSNs-FA/Ly. Figure S4. a The absorption spectra of Ly with different concentrations. b The standard curve of Ly determined by a UV-VIS spectrophotometer. Figure S5. The corresponding surface plot of ICG fluorescence images of MNNG/HOS cells after incubation with GNS@MSNs/ICG, GNS@MSNs-FA/ICG and GNS@MSNs-FA/ICG + free FA for 4h. Figure S6. Cell viability of MNNG/HOS cells after the corresponding treatment for 24 h. Figure S7. Relative protein levels of Bax and Bcl-2 in MNNG/HOS after various treatment. Figure S8. Relative protein levels of Bax and Bcl-2 in MNNG/HOS after various treatment. Figure S9. Relative protein levels of Cyt-c in MNNG/HOS after various treatment. Figure S10. Biosafety evaluation by blood biochemistry test. a Serum levels of ALT (liver function index). b Serum levels of BUN (kidney function index). [file 12951_2021_933_MOESM1_ESM.docx]

**Supporting Information**

**Amplification of** **oxidative stress** **with** **lycorine and g****old-based** **nanocomposite****s** **for** **synergistic cascade cancer therapy**

Hongzhi Hu ^1^^, 3, *^, Wenbo Yang ^1, *^, Zihui Liang^2, *^, Zezhu Zhou^2^, Qingcheng Song^3^, Weijian Liu^1, 3^, Xiangtian Deng^4^, Jian Zhu^4^, Xin Xing^3^, Binglong Zhong^1^, Baichuan Wang^1^, Shangyu Wang^1, #^, Zengwu Shao^1, #^, Yingze Zhang^1, 3, #^

^1^ Department of Orthopaedics, Union Hospital, Tongji Medical College, Huazhong University of Science and Technology, Wuhan 430022, China.

^2^ Collaborative Innovation Center for Advanced Organic Chemical Materials Co-constructed by the Province and Ministry, Hubei University, Wuhan 430062, China

^3^ Department of Orthopaedic Surgery, The Third Hospital of Hebei Medical University, Shijazhuang, 050051, China.

^4^ School of Medicine, Nankai University, Tianjin, 300071, China.


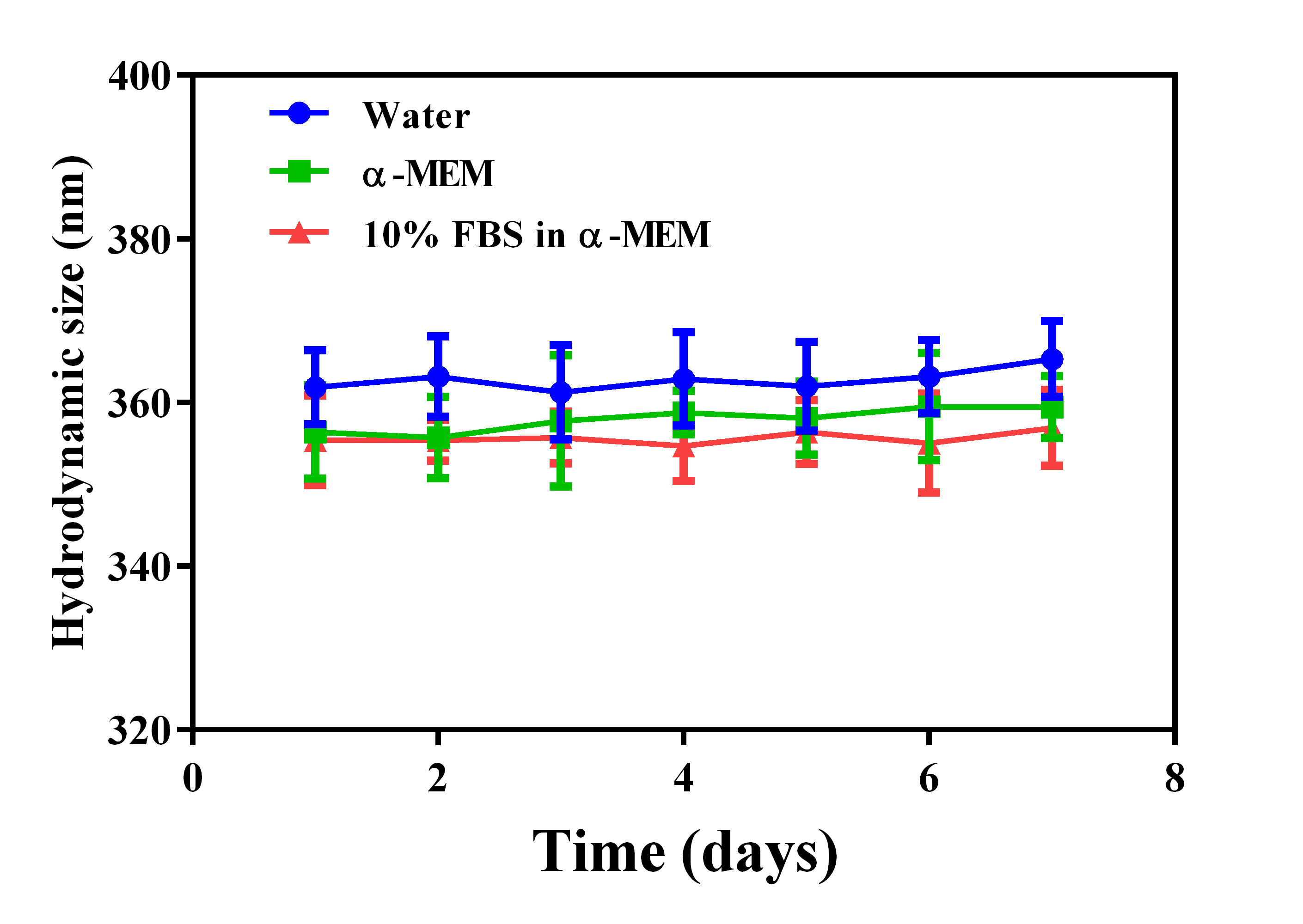


**Figure. S1** Hydrodynamic size variation of the GNS@MSNs-FA/Ly NPs dispersed in water, α-MEM culture medium with or without 10% FBS.


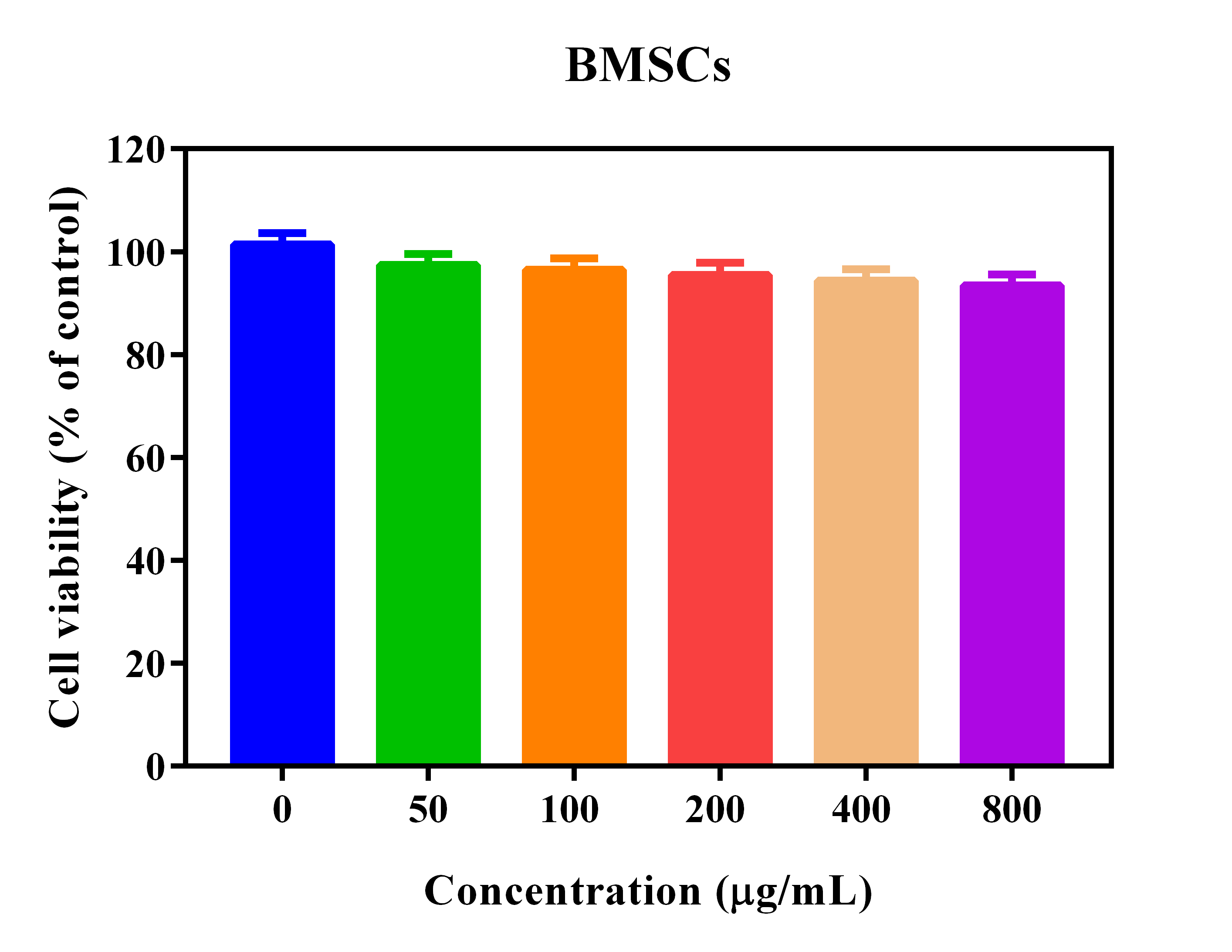


**Figure. S2** Cell viability of BMSCs cells after 24 h of treatments with various concentrations of GNS@MSNs-FA.


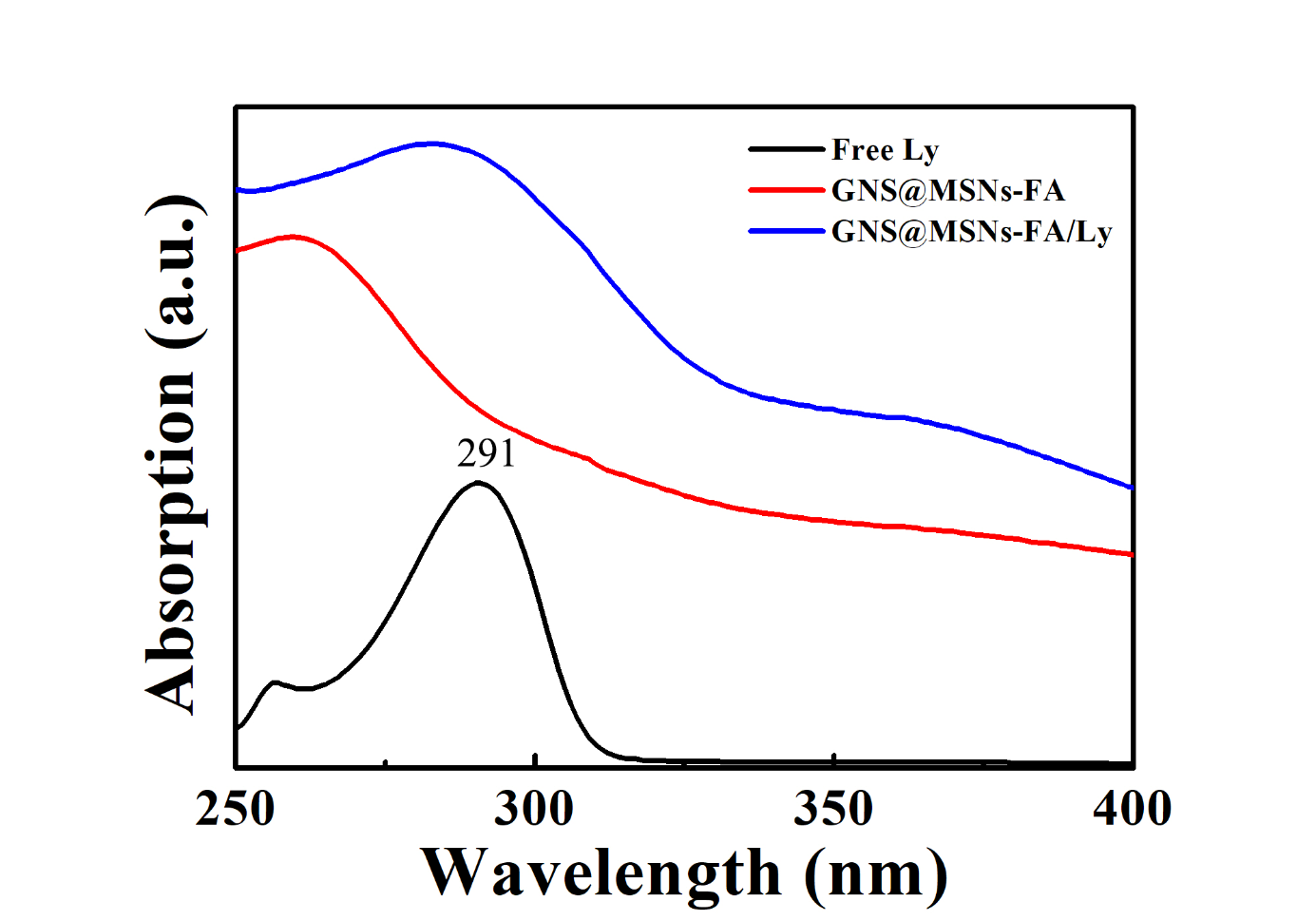


**Figure. S3** UV-Vis spectra of Ly, GNS@MSNs-FA and GNS@MSNs-FA/Ly.


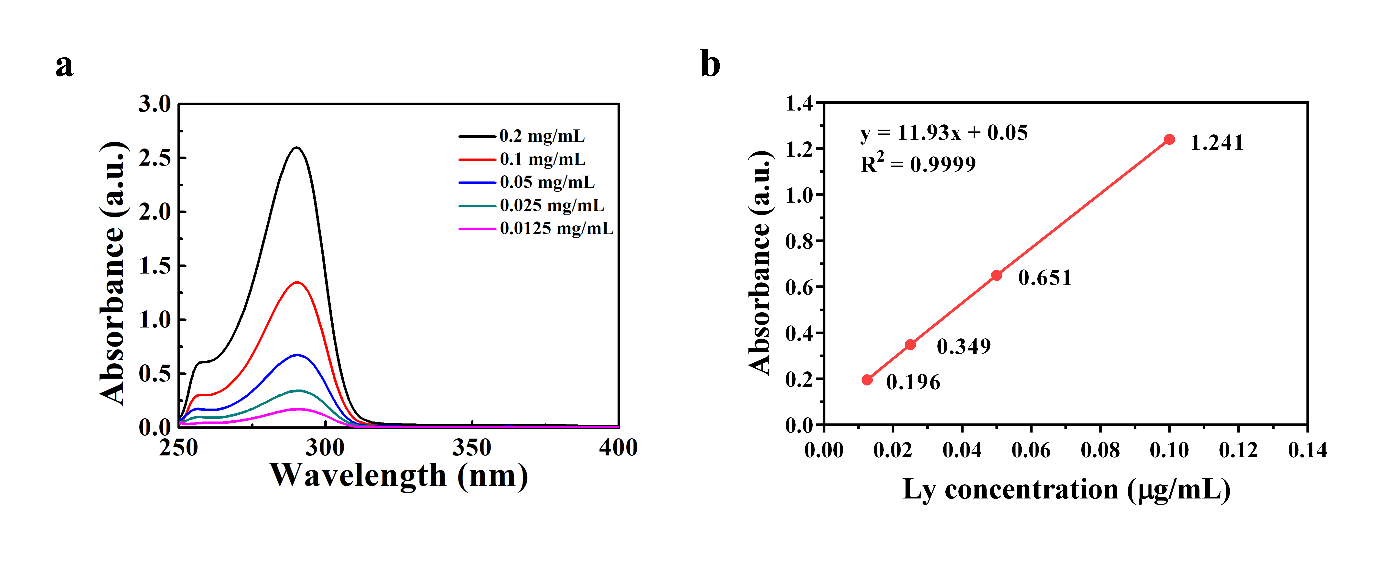


**Figure. S4 a** The absorption spectra of Ly with different concentrations. **b** The standard curve of Ly determined by a UV-VIS spectrophotometer.


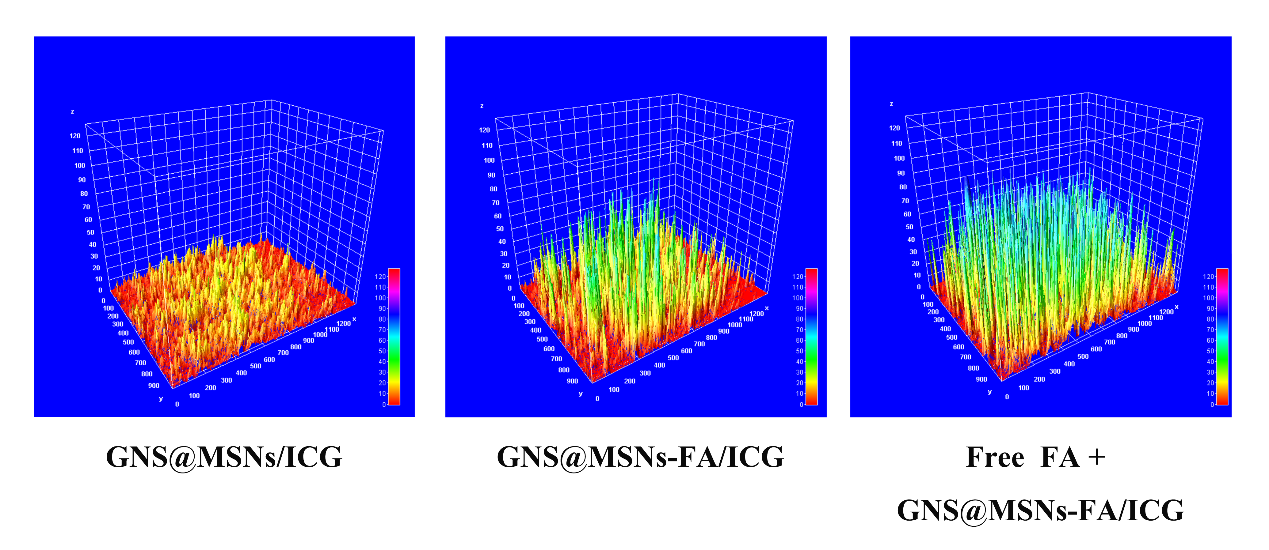


**Figure. S****5** The corresponding surface plot of ICG fluorescence images of MNNG/HOS cells after incubation with GNS@MSNs/ICG, GNS@MSNs-FA/ICG and GNS@MSNs-FA/ICG + free FA for 4h.


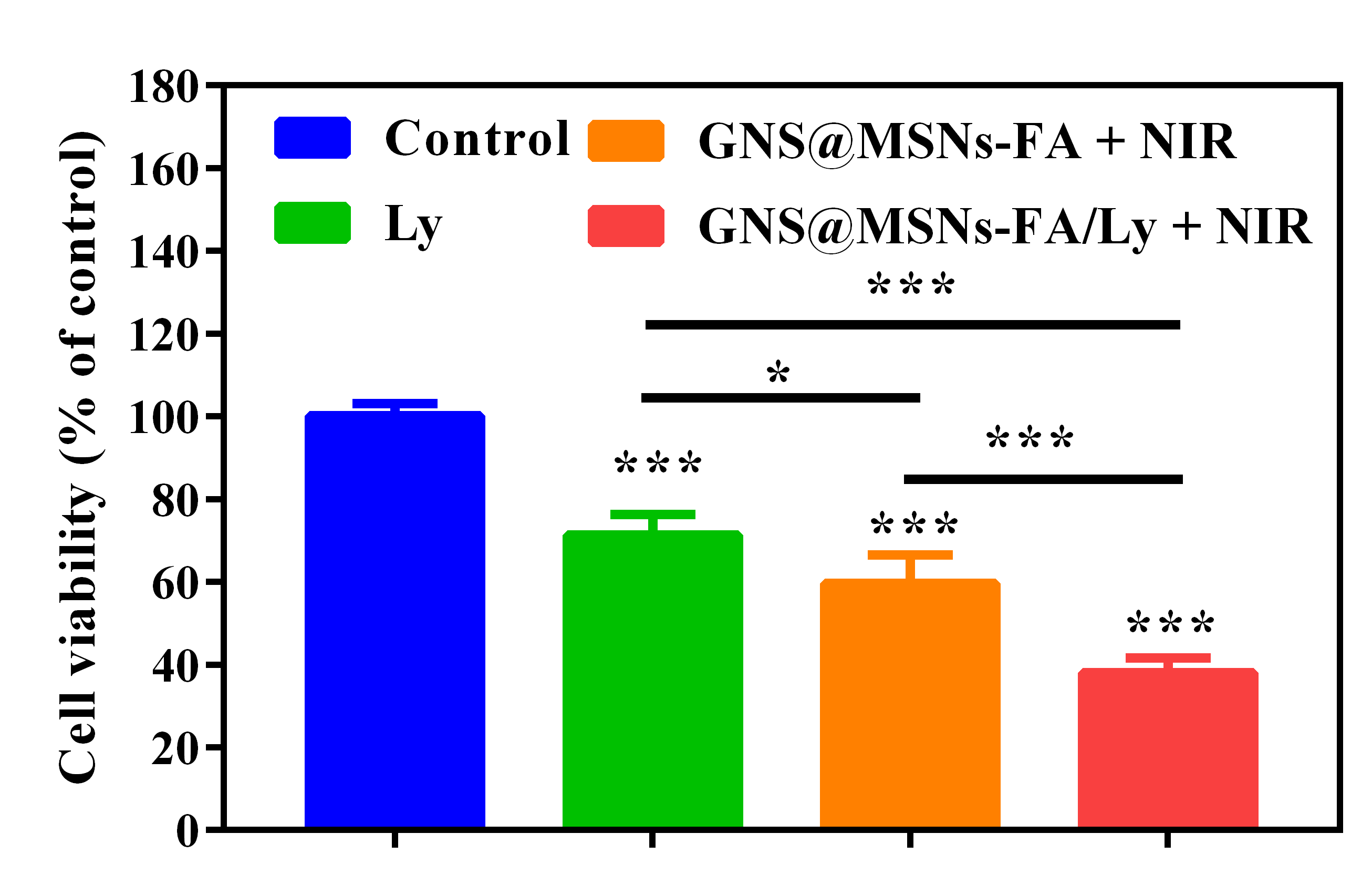


**Figure. S6** Cell viability of MNNG/HOS cells after the corresponding treatment for 24 h.


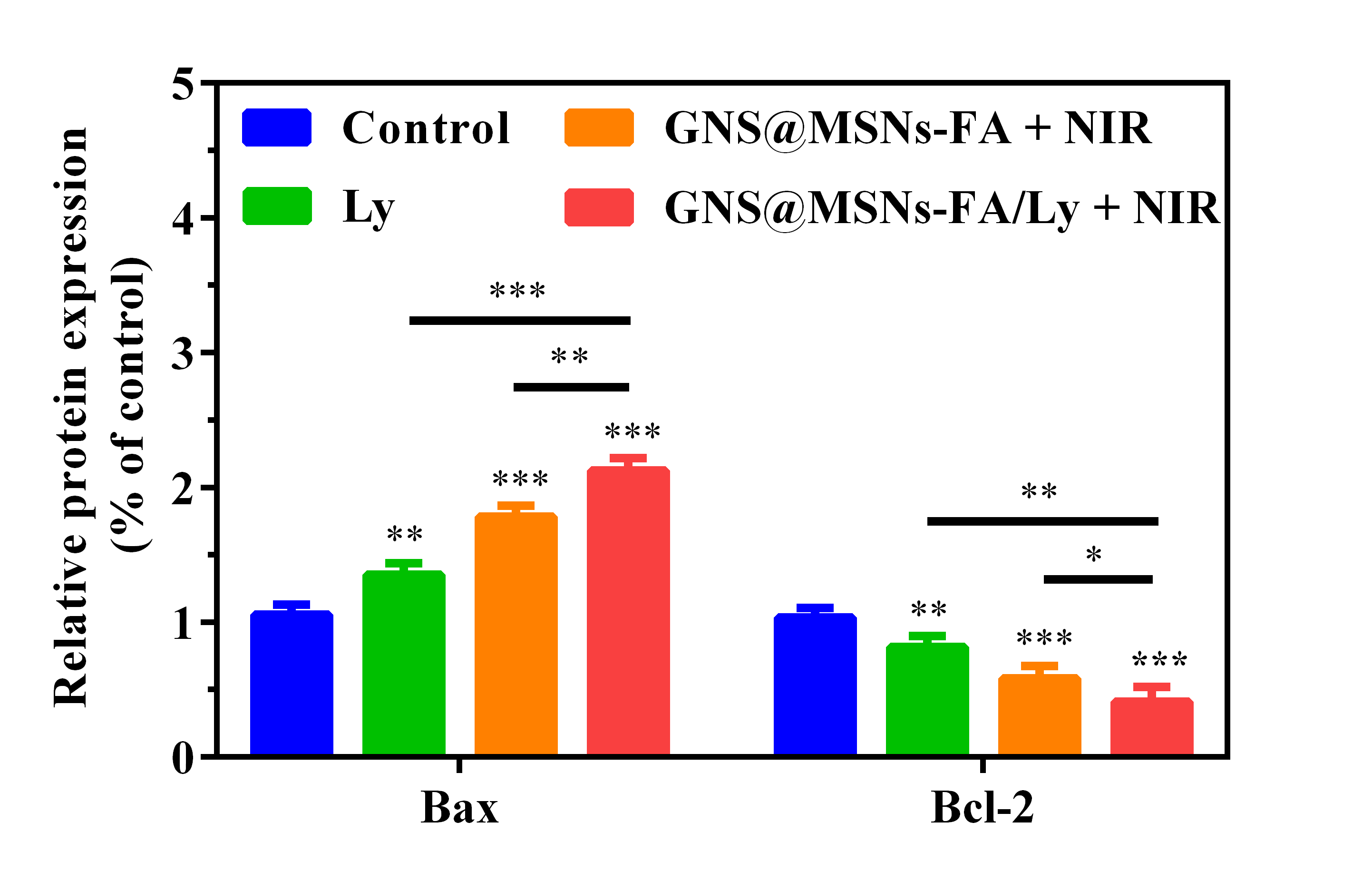


**Figure. S7** Relative protein levels of Bax and Bcl-2 in MNNG/HOS after various treatment.


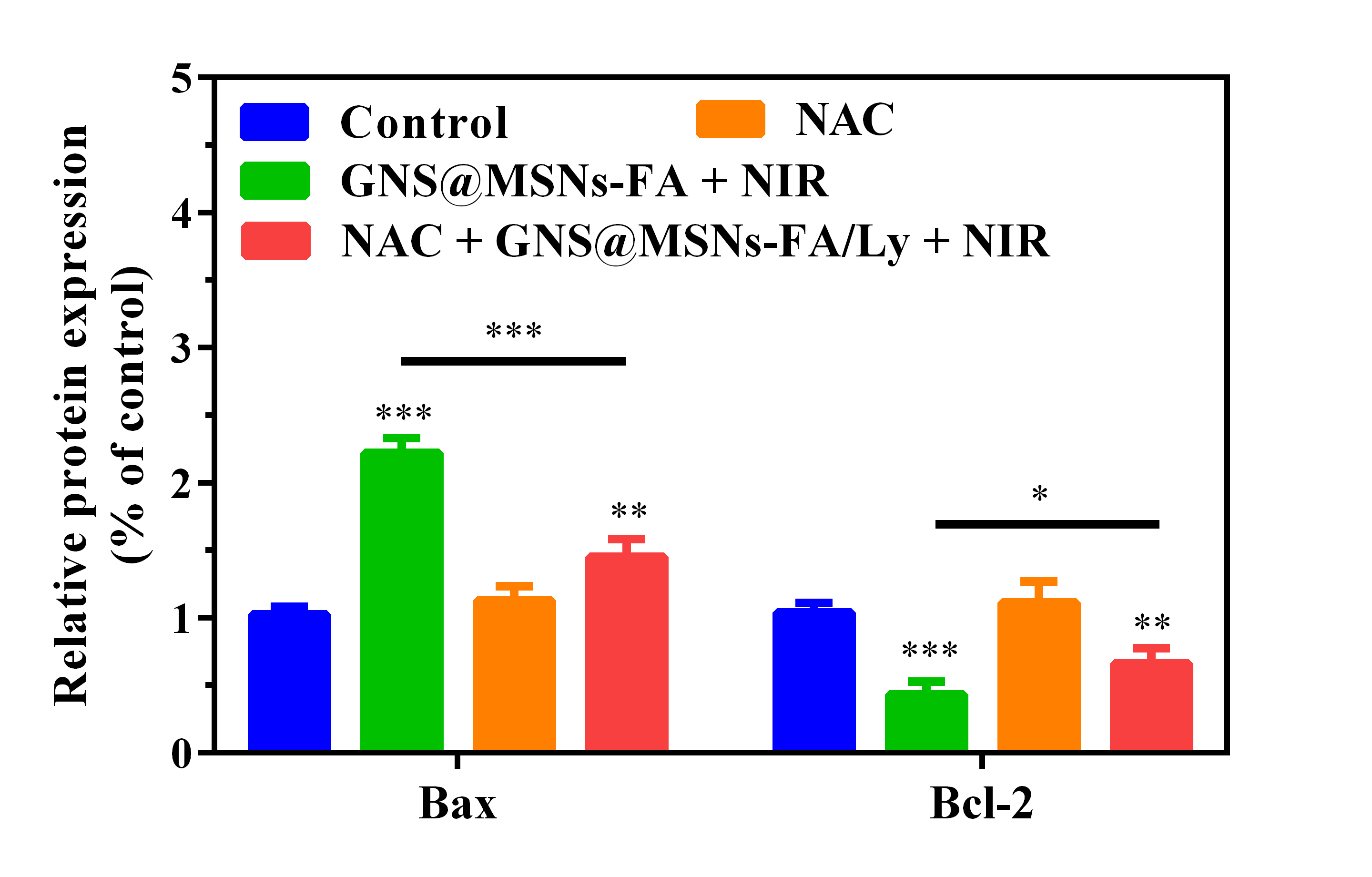


**Figure. S8** Relative protein levels of Bax and Bcl-2 in MNNG/HOS after various treatment.


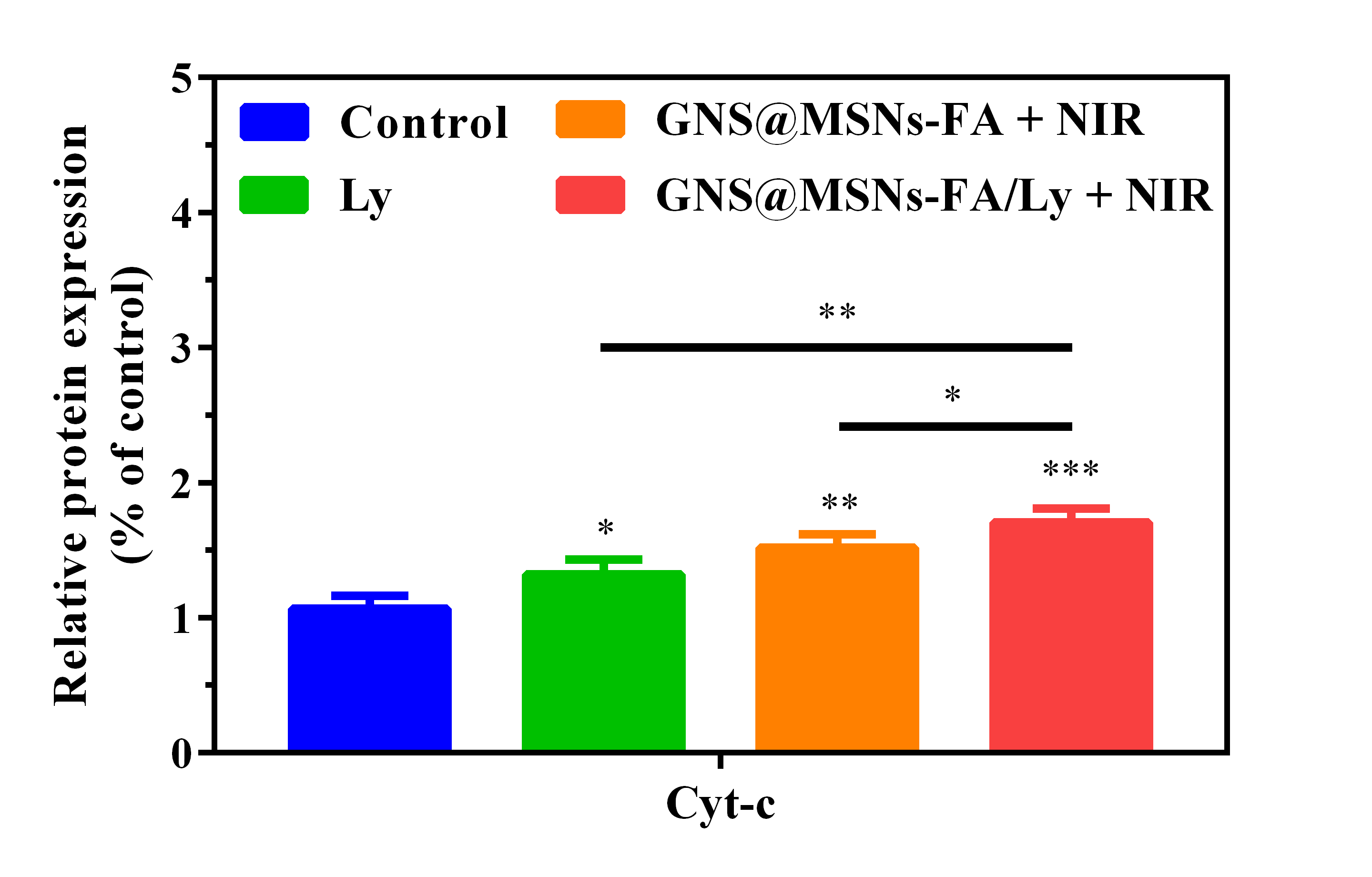


**Figure. S9** Relative protein levels of Cyt-c in MNNG/HOS after various treatment.


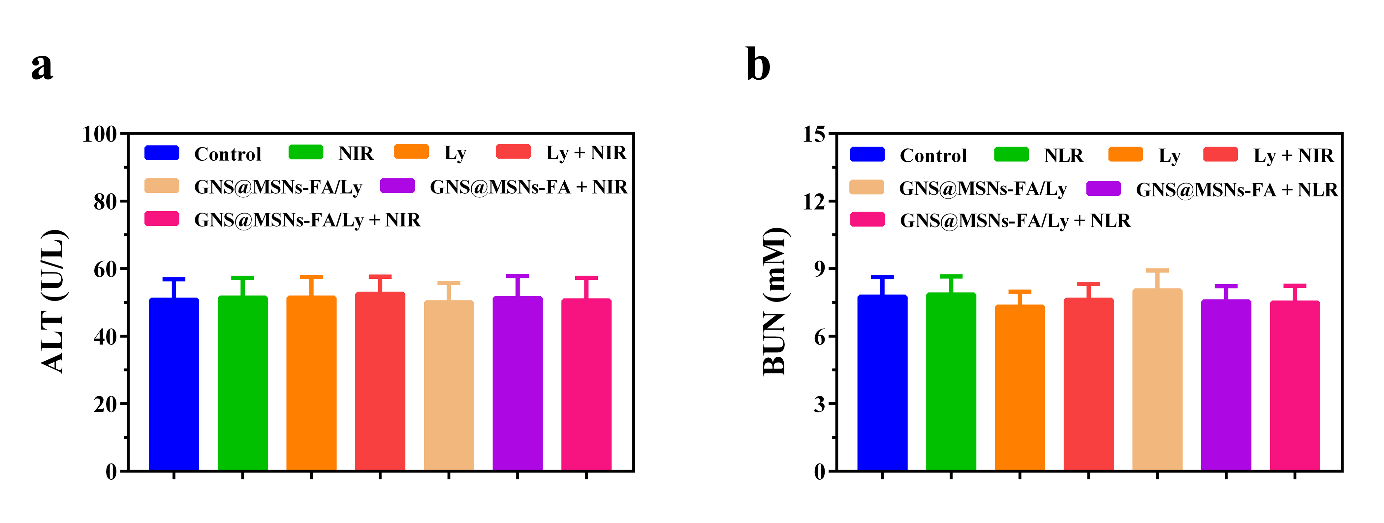


**Fi****gure. S10** Biosafety evaluation by blood biochemistry test. **a** Serum levels of ALT (liver function index). **b** Serum levels of BUN (kidney function index).
